# Supplementary material for: Automatic Segmentation of Drosophila Neural Compartments Using GAL4 Expression Data Reveals Novel Visual Pathways
Source: Curr Biol. 2016 Aug 8;26(15):1943–54. doi: 10.1016/j.cub.2016.05.052 (PMC4985560; doi:10.1016/j.cub.2016.05.052)
Supplement: Document S1. Supplemental Experimental Procedures, Figures S1–S3, and Table S1 [file mmc1.pdf]

Current Biology, Volume *26*

Supplemental Information

Automatic Segmentation of *Drosophila* Neural Compartments Using GAL4 Expression Data Reveals Novel Visual Pathways

Karin Panser, Laszlo Tirian, Florian Schulze, Santiago Villalba, Gregory S.X.E. Jefferis, Katja Bühler, and Andrew D. Straw

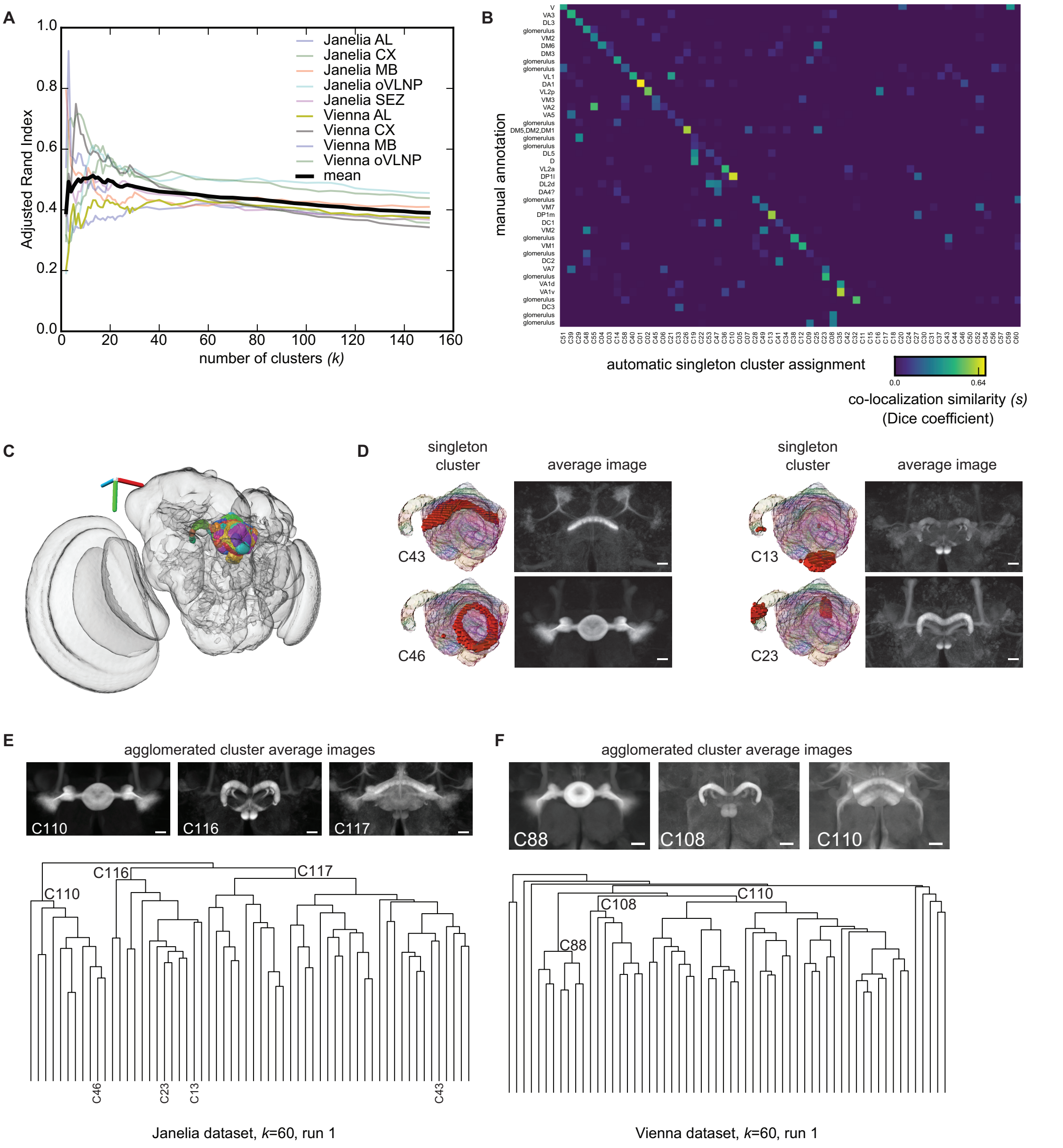

Figure S1. (data related to Figure 1): Evaluation of *k*-medoids clustering for automatically segmenting brain regions into anatomical structures.

(A) Repeatability scores across multiple runs of the *k*-medoids algorithm. The adjusted Rand index, a measure of repeatability, was calculated based on 10 repeated runs of the *k*-medoids algorithm for both datasets and several brain regions.

(B) Colocalization similarity (measured as Dice coefficient *s* on the set of voxels in the manually annotated region and the set in the clustering result) between the Janelia FlyLight dataset and manual assignments using the same 3D template brain. Manual assignments were based on a manually segmented neuropil image. Glomeruli that could not be unambiguously identified were labeled “glomerulus”. (Janelia FlyLight data for the right antennal lobe region, run 1, 6502 voxels, 3462 driver lines, *k* equal 60.)

(C) Automatic segmentation of central complex (CX). 3D axes scale 30 µm.

(D) Individual singleton clusters (left) and average image of strongly expressing driver lines in each cluster with broad driver lines removed (right). Scale bars 20 µm.

(E) Average images from agglomerated clusters (top) and dendrogram of agglomerated hierarchy. Scale bars 20 µm.

(F) As in E, but from the Vienna dataset, *k*=60. Scale bars 20 µm.

Panels C-E: Janelia FlyLight data for CX, run 1, 27598 voxels, 3462 driver lines, *k*=60.

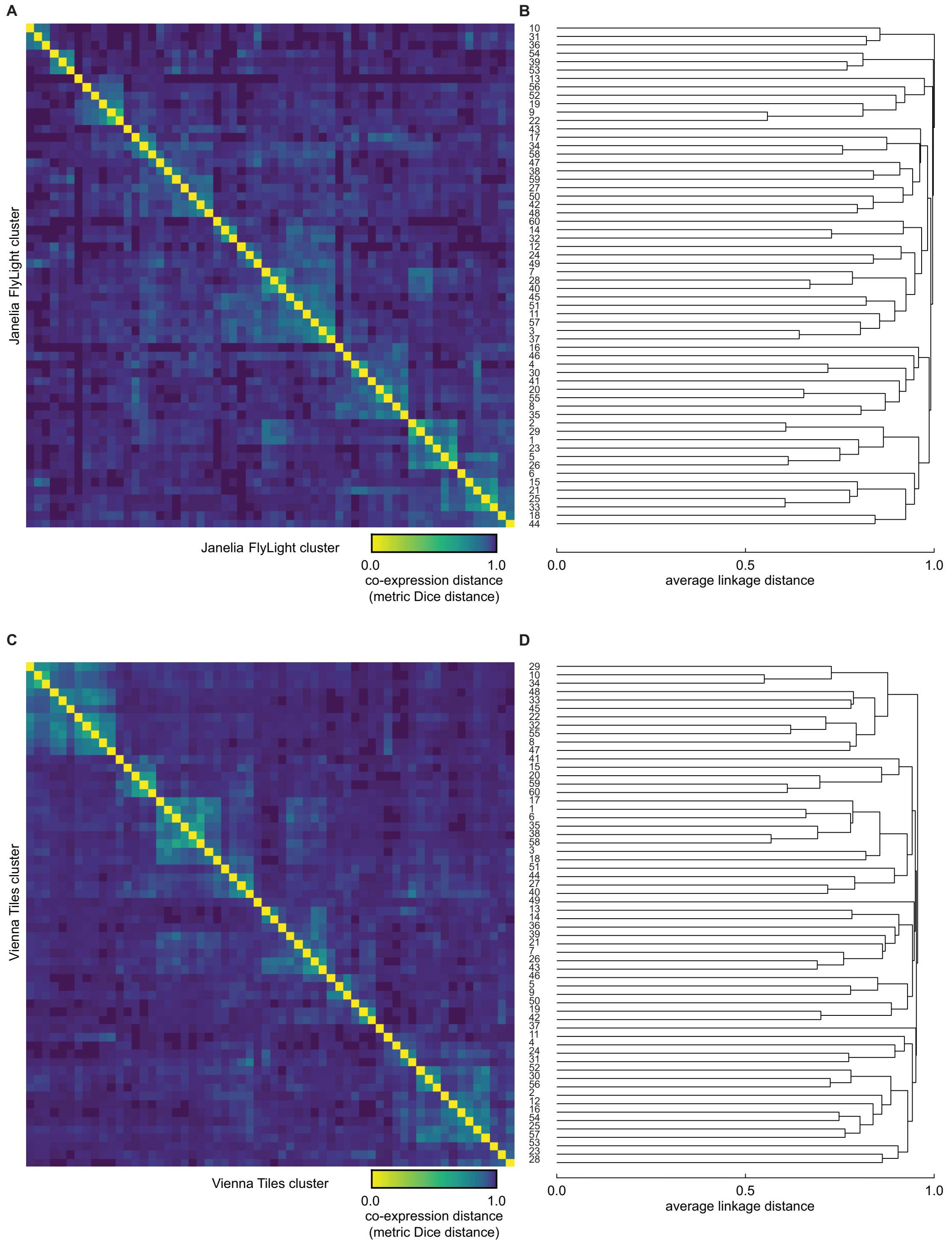

Figure S2. (data related to Figures 2-3): Clustering quality for oVLNP in both datasets.

(A) Quantification of similarity between clusters as measured by voxel-to-voxel co-expression distance ($\sqrt{1-s}$, where s is the Dice coefficient between the two sets of enhancer expression) for each medoid of every cluster of run 1 in the oVLNP region using the Janelia dataset.

(B) Dendrogram of agglomerative hierarchical clustering using average linkage showing a representation of co-expression distance between medoids in the oVLNP region of the Janelia dataset.

(C) Quantification of similarity between clusters as measured by voxel-to-voxel co-expression distance for each medoid of every cluster in the oVLNP region of run 1 the Vienna dataset.

(D) Dendrogram as in B using the Vienna dataset.

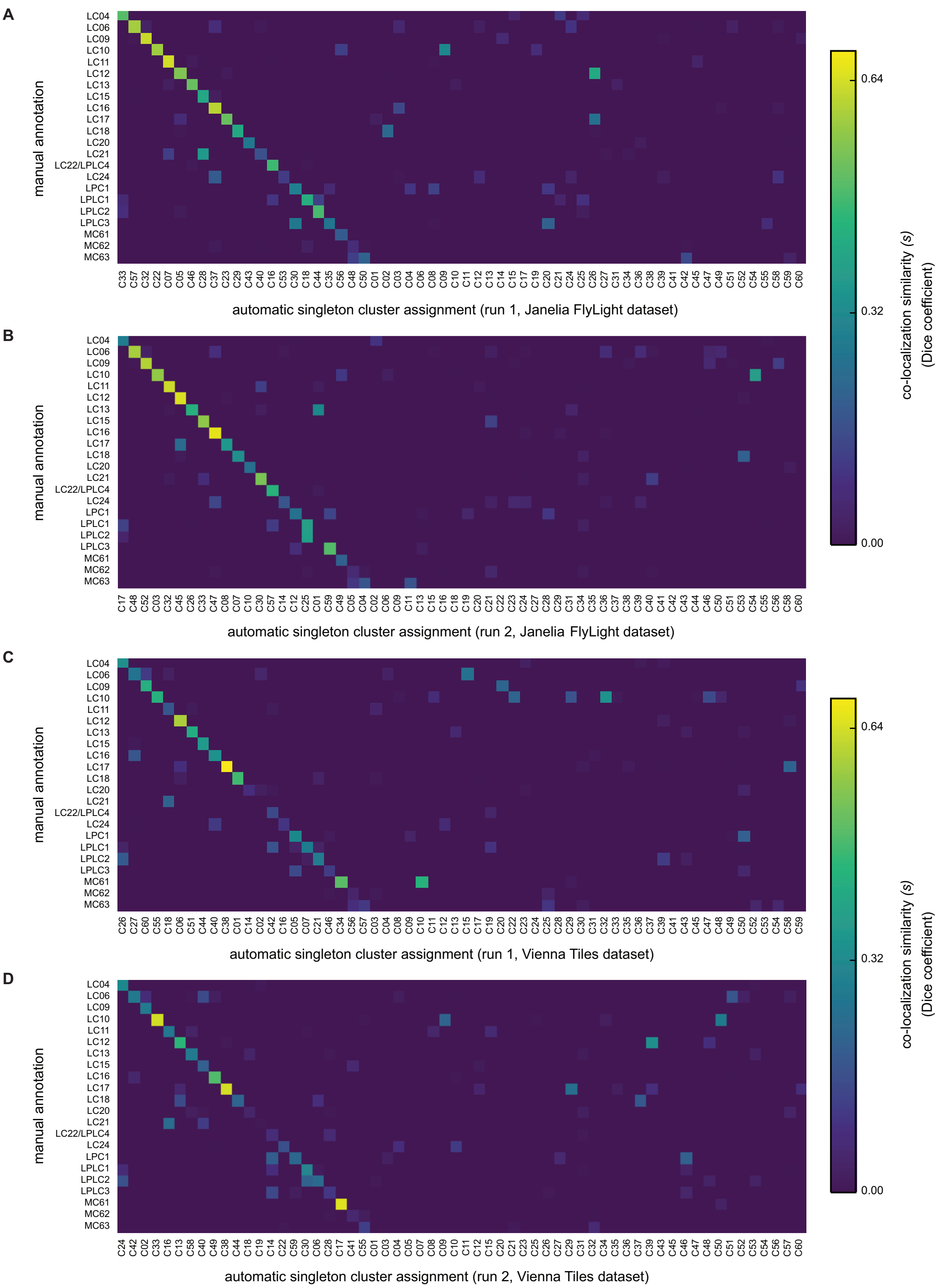

Figure S3. (data related to Figures 2-3): Automatically assigned oVLNP singleton clusters colocalize with manually segmented optic glomeruli, repeated clustering of the same dataset gives similar results, and clustering of different datasets gives similar results.

(A-B) Colocalization similarity (measured as Dice coefficient *s* on the set of voxels in the manually annotated region and the set in the clustering result) between the Janelia FlyLight dataset and manual assignments using the same 3D template brain. (Janelia FlyLight data for oVLNP, 42317 voxels, 3462 driver lines, *k* equal 60.)

(C-D) Colocalization similarity between the Vienna Tiles dataset and manual assignments using the same 3D template brain. (Vienna Tiles data for oVLNP, 13458 voxels, 6022 driver lines, *k* equal 60.)

VPN type	Synonyms	Best enhancers identified for neuron type from Janelia GAL4 library	Best enhancers identified for neuron type from Vienna tiles (VT) GAL4 library	FlyCircuit.tw - Single cell examples for neuron type	Clusters corresponding to optic glomerulus or tract associated with a VPN			
					C (Janelia FlyLight dataset)	C' (Vienna Tiles dataset)	C'' (Janelia FlyLight dataset, 2nd run)	C''' (Vienna Tiles dataset, 2nd run)
LC04	Col A (Mu et al., 2012; Strausfeld & Okamura, 2007; Strausfeld and Hausen, 1977)	GMR26G09, GMR47H03	VT042758, VT046005	Cha-F-000138, Cha-F-200257, Gad1-F-300256	C33, C21, C15, C25	C'26, C'39	C''02, C''17	C'''24
LC06	S4 (Fischbach and Lyly-Hünerberg, 1983)	GMR41C07, GMR22A07	VT006549, VT009855	Cha-F-000039, Gad1-F-400244, Gad1-F-200326	C57	C'27	C''48	C'''42
LC09	S4 (Fischbach and Lyly-Hünerberg, 1983)	GMR71C02, GMR14A11	VT014209, VT005102, VT027704	Cha-F-000028, Gad1-F-700145, Gad1-F-200274	C32, C14	C'59, C'60	C''52, C''56, C''35	C'''02
LC10	S3 (Fischbach and Lyly-Hünerberg, 1983)	GMR22D06, GMR35D04	VT021760, VT043920	Gad1-F-100080, Cha-F-300390, fru-F-800100	C22, C09, C19	C'32, C'55, C'48, C'29	C''03, C''54, C''49, C''06	C'''33, C'''34, C'''50
LC11	L1CN (Mu et al., 2012)	GMR23D02, GMR87B04, GMR51F09, GMR22H02	VT004968, VT008647, VT004967	Cha-F-000153, Cha-F-200132, Gad1-F-300060	C07, C45	C'18	C''32. C''30	C'''16
LC12		GMR59B10, GMR35D04, GMR19G01	VT062247, VT040919	Cha-F-000124, Cha-F-000015, VGlut-F-000056, VGlut-F-400347	C26, C05	C'06	C''45	C'''39, C'''13
LC13		GMR50C10, GMR14A11	VT057283, VT025771	Cha-F-000255, Cha-F-100003, Gad1-F-100040	C46	C'51	C''26, C''01	C'''58
LC14	DC neurons (Hassan et al., 2000)	GMR21H10, GMR12F01, GMR58H11	VT037804	Cha-F-400228, Cha-F-400231, Gad1-F-300016	x	C'03	C''34	C'''08
LC15		GMR42H06, GMR24A02	VT014207, VT047878, VT012320	Cha-F-000361, Cha-F-100351	C28	C'44	C''33, C''21	C'''41, C'''40
LC16		GMR32D04, GMR25G03	VT061079, VT025771	Gad1-F-100202, Cha-F-000316, fru-F-000032, VGlut-F-000603	C37, C03	C'40, C'27	C''47	C'''49
LC17		GMR21B04, GMR65C12	VT034259, VT033301	Cha-F-100017, Cha-F-000004, Gad1-F-000025	C23, C26, C01	C'35, C'38, C'58	C''08, C''45	C'''38, C'''29, C'''35, C'''11, C'''39, C'''60, C'''12
LC18		GMR92B11	VT008183	5-HT1B-F-500016, Cha-F-000333, fru-F-200061, Gad1-F-300054	C29, C02	C'01	C''07, C''53	C'''37, C'''44
LC20		GMR17A04, GMR71G09	VT025718	VGlut-F-200564, VGlut-F-700163, Gad1-F-200101	C43	x	C''10	x
LC21		GMR85F11, GMR25A07	VT014960	Gad1-F-400102, Cha-F-300208	C40, C28, C07	C'18	C''30, C''40	C'''40, C'''16
LC22/LPLC4		GMR24A05	VT058688	LC22: Gad1-F-900022, Cha-F-600134, VGlut-F-500700<br>LPLC4: Gad1-F-200058, Cha-F-200302, Cha-F-200028	C16	C'42, C'19	C''57	C'''14
LC24		GMR20G09	VT038216	Cha-F-000283, Cha-F-200073, Cha-F-400116	C37	C'40	C''47	C'''10
LPLC1	LPL2CN (Mu et al., 2012)	GMR36B06, GMR12G03	VT007767	Cha-F-200219, Cha-F-300035, Gad1-F-400140	C18, C44, C25	C'07	C''25	C'''30
LPLC2		GMR75G12, GMR12E04	VT007194, VT049479	Gad1-F-000300, Cha-F-100287, Cha-F-300111	C44	C'21	C''25	C'''06, C'''30
LPLC3		GMR9C11, GMR49A05	VT044492, VT062624	Cha-F-100027, Cha-F-300004, Gad1-F-200099, fru-F-500009	C35, C55, C20, C30	C'46, C'05, C'09	C''59, C''13, C''19	C'''28, C'''14
LPC1		GMR37G12, GMR77A06, GMR81A05, GMR20A09 (subset)	VT046005	VGlut-F-700361, Cha-F-000272, fru-F-000101	C04, C30, C20	C'05	C''12, C''59, C''19	C'''46
MC61	LC10c (Otsuna & Ito, 2006)	GMR53B08	VT002072, VT021203	Gad1-F-400023, Cha-F-300285, Cha-F-200026,	C56	C'34, C'10	C''49	C'''17
MC62		GMR78G04, GMR85C01	VT062624	none identified	C48	C'56	C''05	x
MC63	VPN-MB1? (Vogt et al., 2016)!	GMR72C11	VT022290, VT008183, VT017001	Cha-F-200103	C42, C48	C'25, C'56	C''04, C''11, C''05	C'''55
Lat		GMR16G04, GMR13E10, GMR85G07, GMR39F04	VT045604, VT014963, VT033613	TH-F-200107, Trh-F-100019, TH-F-100004, Cha-F-300333	C50, C42	C'30, C'52, C'56, C'57	C''04	C'''55

Table S1. (data related to Figure 4): Table with VPN, Clusters, Driver lines, Flycircuit IDs.

Note: MC63 may be synonymous with VPN-MB1 [S3], which was published while this study was under review.

Supplemental Experimental Procedures

Thresholding, Dice similarity, k-Medoids, and Hierarchical Agglomeration

GAL4 expression patterns were transformed into a binary representation in two steps. First, the image is thresholded and second, morphological opening (dilation of the erosion by a 3x3x3 structuring kernel) is applied to reduce clutter. The threshold was chosen so that the resulting mask yielded 1% stained voxels. This simple heuristic was more reliable for the datasets tested compared to other standard automatic thresholding methods.

From the binarized images, the set of expressing lines was assembled for each voxel. Similarity between voxels based on the respective expression set from voxel A and the set from voxel B is computed using Dice's coefficient as $s = \frac{2|A \cap B|}{|A| + |B|}$ where ∩ denotes intersection and |*x*| denotes the number of elements in set *x*. To decrease the effects of registration error and image acquisition noise and to increase the speed of subsequent processing steps, we binned the original image voxel data into larger voxels, using a 3x3x3 nearest-neighbor downsampling. Analysis was performed on specific brain regions (e.g. antennal lobe or oVLNP) defined by a 3D brain atlas of neuropils (included in the supplemental data). Voxels in the bounding cube but not in the defined neuropil were excluded. The k-medoids algorithm [S1] was run in Julia 0.4.0 using JuliaStats Clustering 0.5.0 (see Supplementary file 1). The *k*-medoids was performed on Dice dissimilarity (1-*s*). To agglomerate the medoids, we used the fastcluster package [S2] with Python 2.7.10 using average linkage with metric distance $\sqrt{1-s}$ between medoids.

Initial clustering was performed on a distance matrix found as follows. For each voxel within the analyzed brain region (e.g. antennal lobe or lateral protocerebrum), we calculated the set of driver lines for which GFP expression was higher than a threshold. We used the Dice coefficient (a measure of overlap, see above) to quantify expression similarity between each possible pair of *n* voxels. This *n* x *n* distance matrix was used to group voxels into clusters of similar expression using *k*-medoids clustering, a standard clustering technique (Figure 1A, see Experimental Procedures for details). Clustering with other standard algorithms such as mini-batch *k*-means gave qualitatively similar results, and we focus here on *k*-medoids only for convenience. As typical for clustering algorithms, one parameter controls the number of clusters, and in our case we chose several different values for *k* and evaluated results for different choices and in each of the two independent datasets. Every voxel in the analysis is assigned to exactly one cluster. Neither manual inspection nor calculation of a metric designed to measure clustering repeatability, adjusted Rand index (Figure S1A), showed an obvious optimal value for *k*. Therefore, we chose a value of *k* equal 60 as a number which appeared to provide sufficiently many

clusters to capture important structures at a small scale without producing an overwhelming number. The result of the initial clustering algorithm is the assignment of each voxel in the input brain region to one of k clusters. The second major step, hierarchical clustering, took the cluster centers from the first step and agglomerated these 'singletons' into $2k$-1 clusters.

Evaluating repeatability of clustering

As discussed above, automatic calculation of a measure of repeatability (adjusted Rand index, Figure S1A) found no obvious optimum value of k used in the initial clustering step. Therefore, we sought to gain a more biologically meaningful sense of consistency across multiple runs of the algorithm for $k=60$ by comparing visually the results of manual and automatic segmentations. We did this for the oVLNP with each of four different clustering runs, two from each dataset (Figure S3). The results show that, despite different random number initialization seeds, most optic glomeruli have a strong correspondence with a singleton cluster across repeated runs of the algorithm within and across the two datasets (Vienna Tiles and Janelia FlyLight). This indicates substantial biologically meaningful repeatability within and between datasets at the first clustering step, which agglomeration then structures hierarchically.

Supplemental References

S1. Kaufman, L., and Rousseeuw, P. J. (1987). Clustering by Means of Medoids. In *Statistical Data Analysis Based on the L1 Norm and Related Methods.*

S2. fastcluster: Fast Hierarchical, Agglomerative Clustering Routines for R and Python *Journal of Statistical Software* https://www.jstatsoft.org/article/view/v053i09.

S3. Vogt, K., Aso, Y., Hige, T., Knapek, S., Ichinose, T., Friedrich, A. B., Turner, G. C., Rubin, G. M., and Tanimoto, H. (2016). Direct neural pathways convey distinct visual information to Drosophila mushroom bodies. *eLife 5*, e14009.
