# Supplementary figures and images for: Automatic Segmentation of Drosophila Neural Compartments Using GAL4 Expression Data Reveals Novel Visual Pathways
Source: Curr Biol. 2016 Aug 8;26(15):1943–54. doi: 10.1016/j.cub.2016.05.052 (PMC4985560; doi:10.1016/j.cub.2016.05.052)

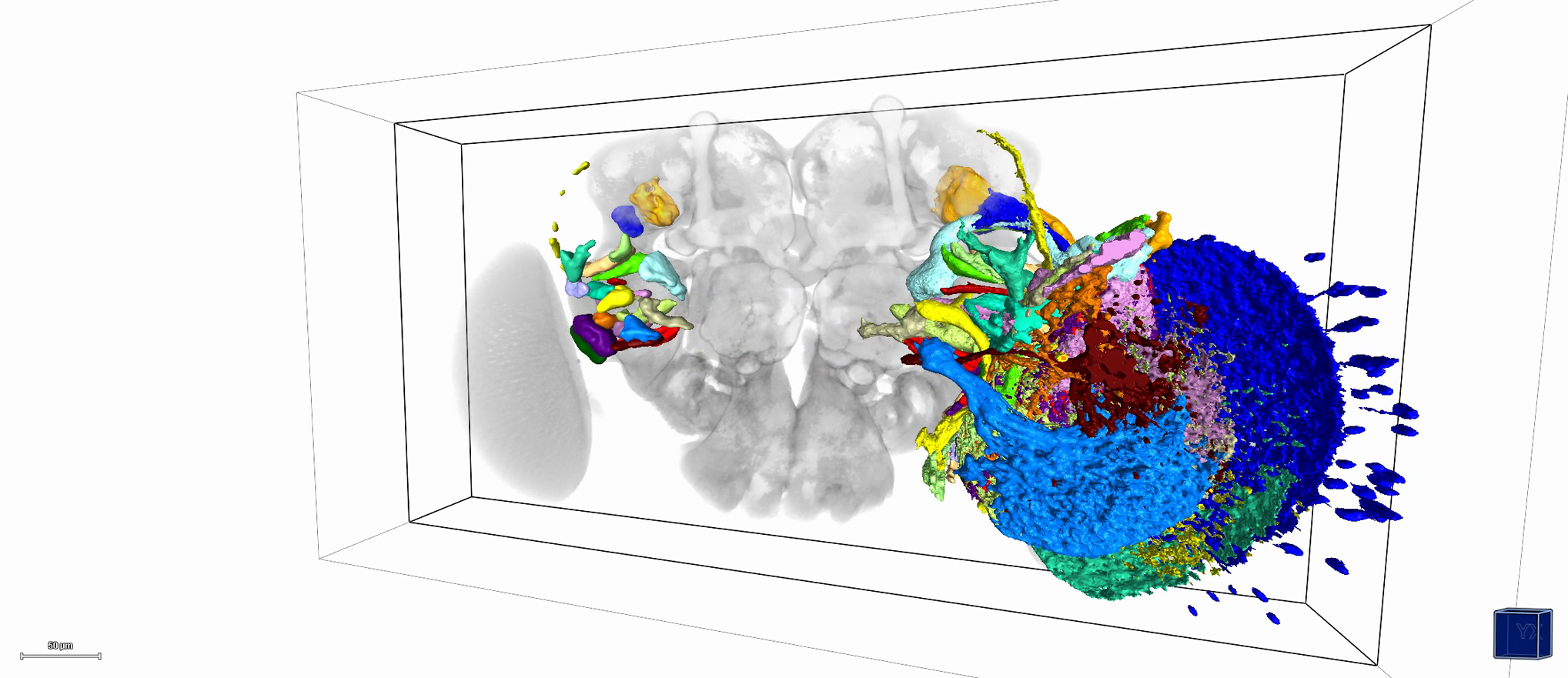

Supplement: Movie S1. Three-Dimensional Location of Manually Segmented Visual Projection Neurons and Optic Glomeruli, Related to Figure 4 — Right: 3D rendering of all identified optic glomeruli registered onto a 3D reference brain. Optic glomeruli were segmented from single-driver confocal images expressing a presynaptic marker (UAS-synaptotagmin::GFP). Left: 3D rendering of visual projection neurons segmented from single-driver confocal images expressing a non-localized cell-membrane marker (UAS-CD8::GFP). [file mmc2.jpg]
