# Supplementary material for: Automatic Segmentation of Drosophila Neural Compartments Using GAL4 Expression Data Reveals Novel Visual Pathways
Source: Curr Biol. 2016 Aug 8;26(15):1943–54. doi: 10.1016/j.cub.2016.05.052 (PMC4985560; doi:10.1016/j.cub.2016.05.052)
Supplement: Document S2. Article plus Supplemental Information [file mmc4.pdf]

Article

Current Biology

Automatic Segmentation of *Drosophila* Neural Compartments Using GAL4 Expression Data Reveals Novel Visual Pathways

Graphical Abstract

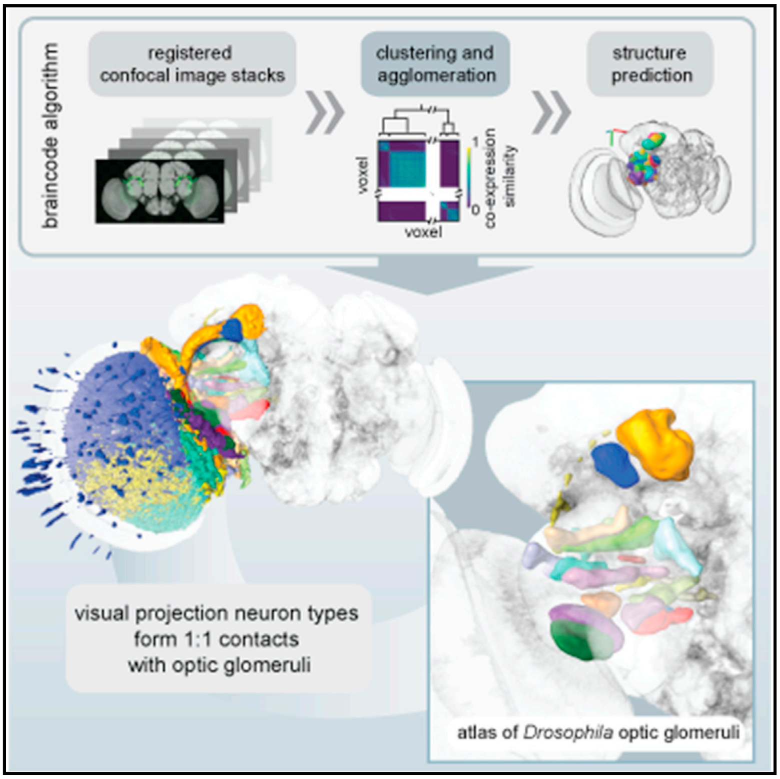

Authors

Karin Panser, Laszlo Tirian, Florian Schulze, Santiago Villalba, Gregory S.X.E. Jefferis, Katja Bühler, Andrew D. Straw

Correspondence

andrew.straw@biologie.uni-freiburg.de

In Brief

In this study, Panser et al. took advantage of recent, genome-wide *Drosophila* enhancer expression datasets consisting of 3D brain image volumes and used clustering to automatically predict brain structures. They validated previously described regions and used novel predictions to make an atlas of the fly optic glomeruli.

Highlights

- Genome-scale enhancer expression patterns can be used to predict brain structure
- Automated clustering of images finds known structures such as olfactory glomeruli
- Results identify GAL4 lines with strong expression in the predicted structures
- We validate novel predictions to reveal previously undescribed optic glomeruli

Panser et al., 2016, Current Biology *26*, 1943–1954
August 8, 2016 © 2016 The Author(s). Published by Elsevier Ltd.
http://dx.doi.org/10.1016/j.cub.2016.05.052

Current Biology
Article

Automatic Segmentation of *Drosophila* Neural Compartments Using GAL4 Expression Data Reveals Novel Visual Pathways

Karin Panser,[1,5] Laszlo Tirian,[1,5,6] Florian Schulze,[2,5] Santiago Villalba,[1] Gregory S.X.E. Jefferis,[3] Katja Bühler,[2] and Andrew D. Straw[1,4,*]
[1]Research Institute of Molecular Pathology (IMP), Vienna Bio-Center, Doktor-Bohr-Gasse 7, 1030 Vienna, Austria
[2]VRVis Zentrum für Virtual Reality und Visualisierung Forschungs, Donau-City-Strasse 1, 1220 Vienna, Austria
[3]Division of Neurobiology, MRC Laboratory of Molecular Biology, Cambridge Biomedical Campus, Francis Crick Avenue, Cambridge CB2 0QH, UK
[4]Department of Neurobiology and Behavior, Institute of Biology I, University of Freiburg, Hauptstrasse 1, 79104 Freiburg, Germany
[5]Co-first author
[6]Present address: Institute of Molecular Biotechnology Austria (IMBA), Vienna Bio-Center, Dr.-Bohr-Gasse 3, 1030 Vienna, Austria
*Correspondence: andrew.straw@biologie.uni-freiburg.de
http://dx.doi.org/10.1016/j.cub.2016.05.052

SUMMARY

Identifying distinct anatomical structures within the brain and developing genetic tools to target them are fundamental steps for understanding brain function. We hypothesize that enhancer expression patterns can be used to automatically identify functional units such as neuropils and fiber tracts. We used two recent, genome-scale *Drosophila* GAL4 libraries and associated confocal image datasets to segment large brain regions into smaller subvolumes. Our results (available at https://strawlab.org/braincode) support this hypothesis because regions with well-known anatomy, namely the antennal lobes and central complex, were automatically segmented into familiar compartments. The basis for the structural assignment is clustering of voxels based on patterns of enhancer expression. These initial clusters are agglomerated to make hierarchical predictions of structure. We applied the algorithm to central brain regions receiving input from the optic lobes. Based on the automated segmentation and manual validation, we can identify and provide promising driver lines for 11 previously identified and 14 novel types of visual projection neurons and their associated optic glomeruli. The same strategy can be used in other brain regions and likely other species, including vertebrates.

INTRODUCTION

A key goal of neuroscientists is to understand brain structure and function and their relation to behavior. Neurogenetic tools that allow easily repeatable targeting of specific cell types now complement classic anatomical techniques, such as Golgi staining, in this pursuit. Such tools have been essential to many advances in the past couple of decades. More recently, genome-scale efforts to develop collections of thousands of *Drosophila* lines in which GAL4 expression is controlled via fragments of genomic DNA containing putative enhancers and repressors [1–3] have already been productively used as the basis for numerous studies.

For many regions of the brain, we lack both a detailed anatomical understanding of the structures present and the ability to reproducibly target specific cell types contained within those structures with genetic tools. For example, extensive work on the visual system of flies such as *Drosophila* [4–15] and other dipteran species [15–20] has shown that the visual projection neurons (VPNs), cells whose projections leave the optic lobes, target structures called “optic glomeruli” in the central brain. Despite this work, the VPNs are incompletely cataloged and no systematic map of the optic glomeruli is available. This region is particularly interesting because the VPNs are an information bottleneck; visual information from the cell-rich optic lobes must pass through the VPNs—with small numbers of cell types and absolute cell counts—before reaching the central brain, where it can influence behavior. For example, in the stalk-eyed fly *Cytrodiopsis whitei*, the optic nerve, containing the VPNs, contains about 6,000 axons [21], and the number of VPN types in *Drosophila* is thought to number about 50 [14]. One suggestion is that optic glomeruli process visual features in a way analogous to olfactory glomeruli in the antennal lobe [13, 16, 19, 20], namely that VPNs of a single type project to a single glomerulus, which receives input from that VPN type in a one-to-one mapping. Each VPN type and corresponding glomerulus may carry information about a specific visual feature, and the array of glomeruli process information so the animal can respond appropriately [11, 13, 16, 19, 20]. As it has been with the *Drosophila* olfactory system, genetic access to VPN cell types and other cell types innervating the optic glomeruli will be useful in elucidating visual circuit function.

Similarly, other regions of “terra incognita,” brain regions that remain largely undescribed, exist even within some of the best-studied brains, including flies and humans [12, 22]. An automatic approach to discover functional units, such as nuclei or axon

© 2016 The Author(s). Published by Elsevier Ltd.
This is an open access article under the CC BY license (http://creativecommons.org/licenses/by/4.0/).

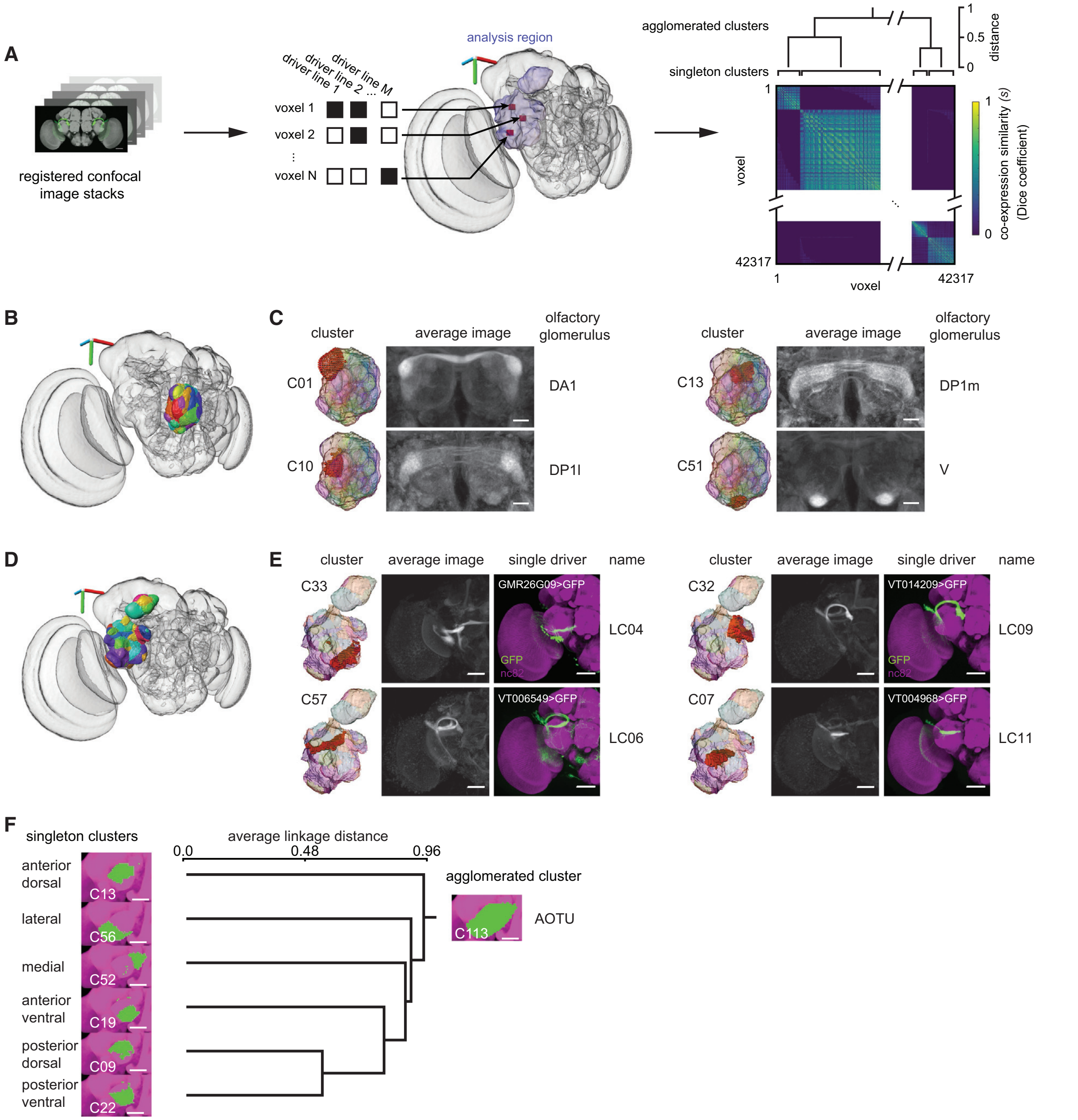

Figure 1. Automatic Segmentation of a Brain Region into Domains Sharing Common Enhancer Profiles

(A) Thousands of registered confocal image stacks from the Janelia FlyLight and Vienna Tiles projects were used. Within an analyzed brain region (purple outline), a list of driver lines driving expression was compiled for each voxel. Voxel-to-voxel similarity *s* was computed using the Dice coefficient, a measure of overlap, and *k*-medoids was used to cluster groups of voxels of putative functional units. These singleton clusters were then agglomerated into a hierarchy.

(B) Automatic segmentation of the antennal lobe (AL). The three-dimensional axis scale is 40 μm in lateral (red), dorsal-ventral (green), and anterior-posterior (blue).

(C) Individual clusters (left), average images of strongly expressing driver lines with broad driver lines removed (middle), and manually assigned corresponding olfactory glomeruli (right). Scale bars, 20 μm.

(D) Automatic segmentation of the optic ventrolateral neuropil (oVLNP). Three-dimensional axis scale, 40 μm.

(E) Individual clusters (left), average images of strongly expressing driver lines with broad driver lines removed (middle), and selected driver lines and previously identified visual projection neuron names (right). Scale bars, 50 μm.

(F) Selected subtree of the agglomerative clustering of the oVLNP results showing z projections of the singleton clusters (left), dendrogram (middle), and top-level agglomeration (right) of the anterior optic tubercle (AOTU). Scale bars, 25 μm.

(legend continued on next page)

tracts, and to suggest genetic lines for targeting these regions would be useful. When compared to the antennal lobes, mushroom bodies, and central complex, much of the *Drosophila* brain is less highly structured, leading to an impression of diffuse neuropils [12]. Several projects have made use of clonal analyses in which rare stochastic genetic events isolating a small number of neurons are assembled to allow reconstructing specific cell types and brain structures [12, 23–28]. Other efforts combine electron microscopy with serial reconstruction to produce detailed connectomic data [29–32]. Despite their utility at revealing brain structure, substantial effort is required to correlate results from these approaches with cell-type-specific genetically encoded markers [33], and thus the results cannot be directly used to identify promising driver lines for subsequent study.

In this study, we used image data from recent *Drosophila* GAL4 collections to automatically identify structure within the fly brain and to identify driver lines targeting these regions. Our approach was based on the hypothesis that multiple locations within a particular nucleus, glomerulus, or axon tract would have patterns of genetic activity, such as gene expression or enhancer activation, more similar to each other than to locations within other structures. RNA expression patterns in mouse [34–37] and human brains [38–41] show this to be true at a relatively coarse spatial scale—sets of genes expressed in, for example, cortex or cerebellum are characteristic for those regions across different individuals. Given that enhancers have more specific expression patterns than the genes they regulate [2], we hypothesized that using enhancers, rather than genes, would enable parcellation of brain regions on a smaller scale. By clustering GFP patterns driven by enhancer-containing genomic fragments, we identified putative functional units. Our results show that, indeed, patterns of genomic-fragment-driven expression can be used to automatically extract brain structure. Many structures of the well-understood *Drosophila* antennal lobes and central complex were automatically found by our method. We further show that this method predicts multiple optic glomeruli and that manual validation confirms the existence and shape of these structural elements. Furthermore, this method highlights existing genetic driver lines likely to be useful for studies of localized neural function.

RESULTS

Segmentation Based on Patterns of Genomic Fragment Co-expression

Our approach to segmenting brain regions into putative "functional units" is based on the idea that multiple locations within such a structure—a brain nucleus, glomerulus, or axon tract, for example—are more similar in their set of active enhancers than locations within other structures. We made use of brain image collections from recent *Drosophila* genomic fragment GAL4 libraries, and the overall strategy was to use conventional clustering and agglomeration techniques on GAL4-driven expression data to parcellate a brain region (e.g., antennal lobe or lateral protocerebrum) into a number of smaller putative functional units (e.g., individual olfactory or optic glomeruli) based on enhancer activity and thus, ultimately, on the genetic code (Figure 1A). This approach divides the brain into distinct, hierarchically organized regions, each likely innervated by multiple cell types. A cluster—a set of voxels—is a distinct concept from a cell type. Whereas local interneurons might be confined to particular clusters, other cell types extend through multiple clusters and into more distant brain regions. Clusters are predictions of functional units in the *Drosophila* brain. Because the strategy links the nucleotide sequence within genomic fragments to specific brain regions, we named it "Braincode." The results can be interactively viewed at https://strawlab.org/braincode.

If our hypothesis—that functional units can be automatically segmented using patterns of enhancer activity and, more specifically, that the clusters identified correspond to genuine anatomical structures—is correct, we can make several predictions. First, despite physical distance not being used as input to the clustering algorithm, we would expect clusters to be spatially compact rather than consisting of, for example, individual voxels scattered throughout the volume. Second, we would expect that for a bilaterally symmetric brain, a given cluster should consist of voxels in mirror-symmetric positions. Third, when clustering is used to segment regions that are already well understood, the shape, size, and location of the automatically found clusters should match the known structures. Fourth, when clustering is performed on different datasets (e.g., Janelia FlyLight versus Vienna Tiles), we expect similar segmentations because the underlying identity of the functional units should dominate the results.

Automatic Segmentation of the Antennal Lobes

To test these expectations, we examined the Braincode results from the antennal lobe (AL) and central complex (CX) (Figure 1; Figure S1). We first examined the singleton clusters—clusters directly from the *k*-medoids algorithm prior to hierarchical agglomeration. As shown, the resulting clusters were compact shapes similar in appearance to the known olfactory glomeruli [42–44] filling the volume of the AL (Figures 1B and 1C).

Individual clusters were highlighted (Figures 1C and 1E, left columns) and used to look at the individual GAL4 lines that have particularly high expression within a given cluster (see https://strawlab.org/braincode) or to take an average of all confocal image stacks from GAL4 lines expressing strongly in a particular cluster, but not elsewhere in the target brain region (Figures 1C and 1E, middle columns). Although our input brain region was the right AL, the average image stacks show a high level of symmetry across the midline. Furthermore, we compared the "ground truth" from manually segmented glomeruli based on a neuropil marker (nc82 antibody) with the results of the initial *k*-medoids clustering. A large fraction of voxels were shared in both datasets (Figure S1B). In a subsequent manual step, we used these correspondences to identify automatically extracted

(A and D–F) Janelia FlyLight data for the oVLNP region defined as the posterior lateral protocerebrum (PLP), posterior ventrolateral protocerebrum (PVLP), and AOTU, run 1, 42,317 voxels, 3,462 driver lines, $k = 60$.
(B and C) Janelia FlyLight data for the right AL, run 1, 23,769 voxels, 3,462 driver lines, $k = 60$.
See also Figure S1.

clusters as specific olfactory glomeruli (Figure 1C, right columns). With $k = 60$ and 43 manually segmented glomeruli, we expected some glomeruli to be split into multiple clusters and, indeed, in some cases, the two automatically determined clusters split a single glomerulus. For example, DM6 was split into singletons C04 and C25, which the agglomeration step joined into agglomerated cluster C74 (Figure S1B). When the same analysis was performed on an entirely independent dataset (Vienna Tiles), the results were qualitatively similar (see the Supplemental Information and https://strawlab.org/braincode).

Central Complex and Other Regions

We performed further clustering on both relatively well understood brain regions and the terra incognita of diffuse neuropils. The central complex has been the focus of substantial work [45–48], and was recently described in detail using split-GAL4 lines and manual annotation [49]. The Braincode algorithm automatically identified many of the prominent structures within this brain region (Figures S1C–S1F). For example, singleton clusters include individual shells of the ellipsoid body neurons, individual layers of the fan-shaped body, and distinct regions of the protocerebral bridge (Figures S1C and S1D). After agglomerating such singleton clusters, we consistently found top-level hierarchies with the major CX substructures (ellipsoid body, fan-shaped body, and protocerebral bridge and noduli; Figures S1E and S1F). In this case, our input brain region spanned the midline to cover the entire CX region and, consistent with expectations for a working algorithm, the clustering results are symmetric across the midline.

The results on the antennal lobe and central complex, both well-studied brain regions, support the idea that patterns of expression can indeed be used to identify functional units and that the Braincode algorithm is capable of automatically proposing brain segmentations of biologically meaningful subregions.

Interpreting Results from Automatic Clustering

Any clustering algorithm has a parameter that (implicitly or explicitly) controls the number of resulting clusters. How should this parameter be set? Ideally, an inherent clustering is easy to identify within the data and trivial for an automatic algorithm to extract. Often, however, and we believe this applies here, exact distinctions are unclear. Clustering algorithms can create classifications different from experts, but experts themselves often disagree due to debates in which "lumpers" argue that differences are insignificant and only obscure a more important deeper unity and "splitters" argue that the differences seen reflect important distinctions. Because we do not expect automated clustering to replace expert reasoning, we instead chose to bias our initial clustering to many clusters, but not overwhelmingly many, and then we structured the results with hierarchical agglomeration for later lumping or splitting.

Due to our interest in the visual system, we evaluated the clustering results in the posterior ventrolateral protocerebrum (PVLP), posterior lateral protocerebrum (PLP), and anterior optic tubercle (AOTU), relatively diffuse neuropils to which the majority of outputs from the medulla and lobula neuropils within the optic lobes project [14, 17, 18]. We call the union of these regions the "optic ventrolateral neuropil" (oVLNP) and note a similar area is called the "optic glomerular complex" in other studies [13, 16, 19]. Dendrograms showing the hierarchical structure from our analysis are shown in Figure S2. Some clusters identified as distinct have little co-expression distance between them and thus might result from excessive splitting, and are the first agglomerations in the hierarchical structure. Conversely, evidence of potential lumping comes from cases such as only one singleton cluster (e.g., C37 in Janelia dataset run 1; Figure S3A) being found for the optic glomeruli innervated by the lobula columnar (LC)16 and LC24 VPN types, despite the fact that manual segmentations of their associated optic glomeruli showed that these project to anatomically distinct (but adjacent) regions (Figures 2B and 2H).

One example that highlights the utility of hierarchical agglomeration to deal with lumping and splitting is the anterior optic tubercle. This area is known in *Drosophila* [14], blow flies [19], honey bee [50], and locust [51] to have several internal compartments. As seen repeatedly in clusterings with different initial random seeds and from both datasets, a single agglomeration typically arises for the entire AOTU, such as with C113 in run 1 of the Janelia FlyLight dataset (Figure 1F). Clear subunits, such as the medial AOTU (C52) and lateral AOTU (C56), are seen, as well as several units within the central AOTU. For example, singleton clusters C09 and C22 correspond to posterior dorsal and posterior ventral parts of the central AOTU, respectively (Figure 1F), and the LC10 neuron type projects to both clusters. Although LC10 subtypes—with distinct morphology and with inputs from distinct layers of the lobula—have been identified that target these regions preferentially [14, 52], our results—separate clusters but low distance as seen in the dendrogram (Figure S2)—suggest that there is relatively little co-expression difference between these regions of the central AOTU. Indeed, after searching through the list of driver lines with substantial expression in C22, we could find only a single driver line, GMR22A07-GAL4, that drove strong expression in a VPN targeting this region and had specificity for Otsuna and Ito's [14] LC10a subtype, but not LC10b. It may be tempting to conclude that the central AOTU was erroneously split by the clustering algorithm, yet the existence of distinct LC10 subtypes suggests there may be genuine, if small, distinctions between these regions. We suggest that the LC10 neuron type and the central AOTU region exemplify the lumping-versus-splitting problem and that hierarchical agglomeration is a practical solution. It is possible that further data, such as detailed studies on LC10-subtype morphology and molecular expression, may resolve the issue. For now, subdividing large brain regions, initially with *k*-medoids clustering and then agglomerating these results into a hierarchical structure, provides a way to reduce complexity when attempting to understand brain structure.

In sum, the automatic hierarchical segmentations produced by Braincode can be used as a starting point for providing hypotheses regarding brain structure and relevant driver lines. The results can be investigated in greater detail, as we have done below for the visual system.

Optic Glomeruli

By analogy to the antennal lobes, where a single glomerulus processes the output of a single type of olfactory sensory neuron, it is proposed that a single VPN type projects to a single optic glomerulus and encodes a single visual feature [13]. As

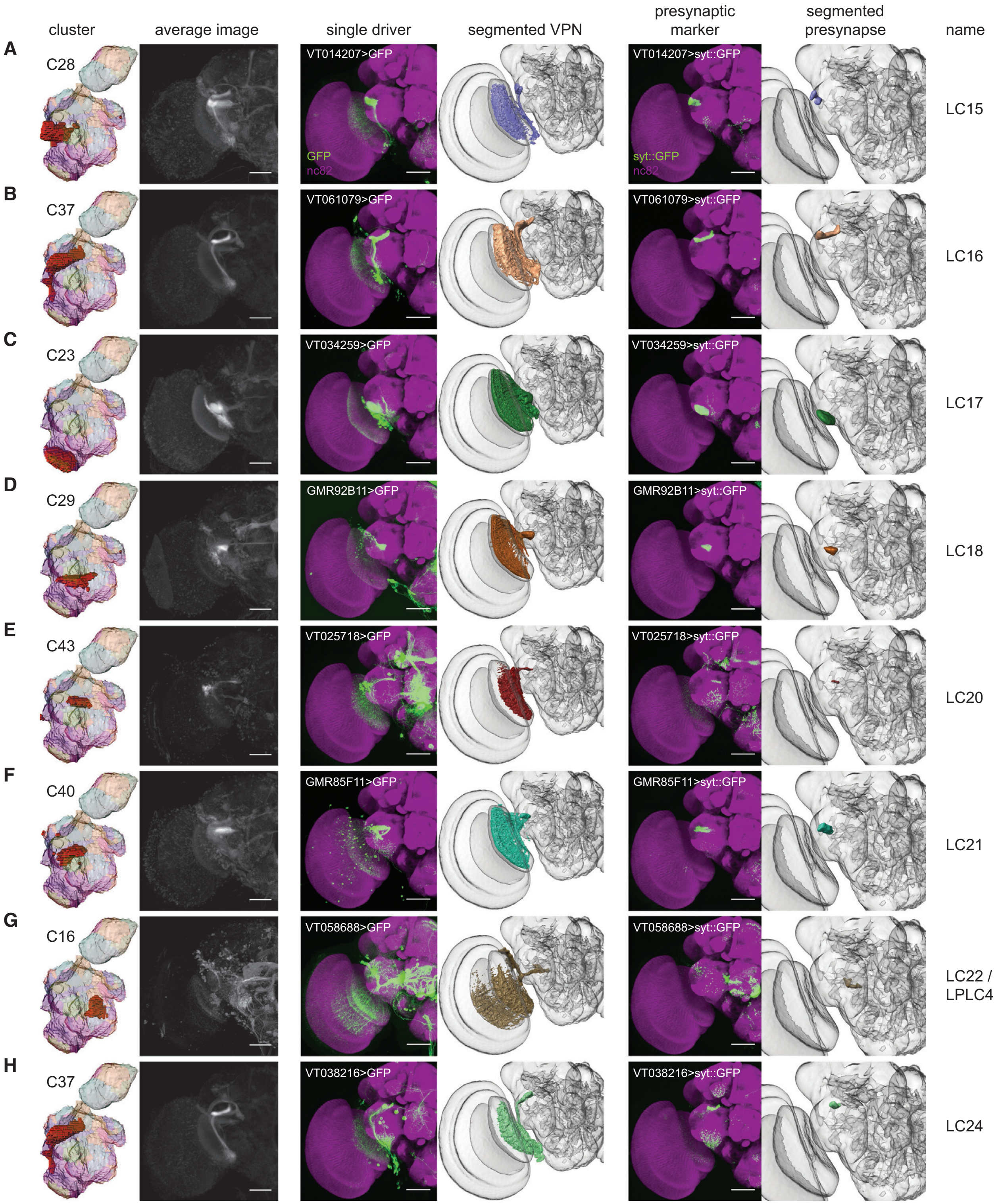

Figure 2. Automatic Segmentation Reveals Clusters that Correspond to Optic Glomeruli Associated with Newly Identified LC-type Visual Projection Neurons

Individual clusters, average images, selected driver lines, 3D segmentations of a particular VPN type, the presynaptic marker (UAS-synaptotagmin::GFP) expressed by a single driver, and 3D segmentation of the presynaptic region to define the optic glomerulus (A–H). Janelia FlyLight data for the oVLNP, run 1, 42,317 voxels, 3,462 driver lines, k = 60. Scale bars, 50 μm. See also Figures S2 and S3.

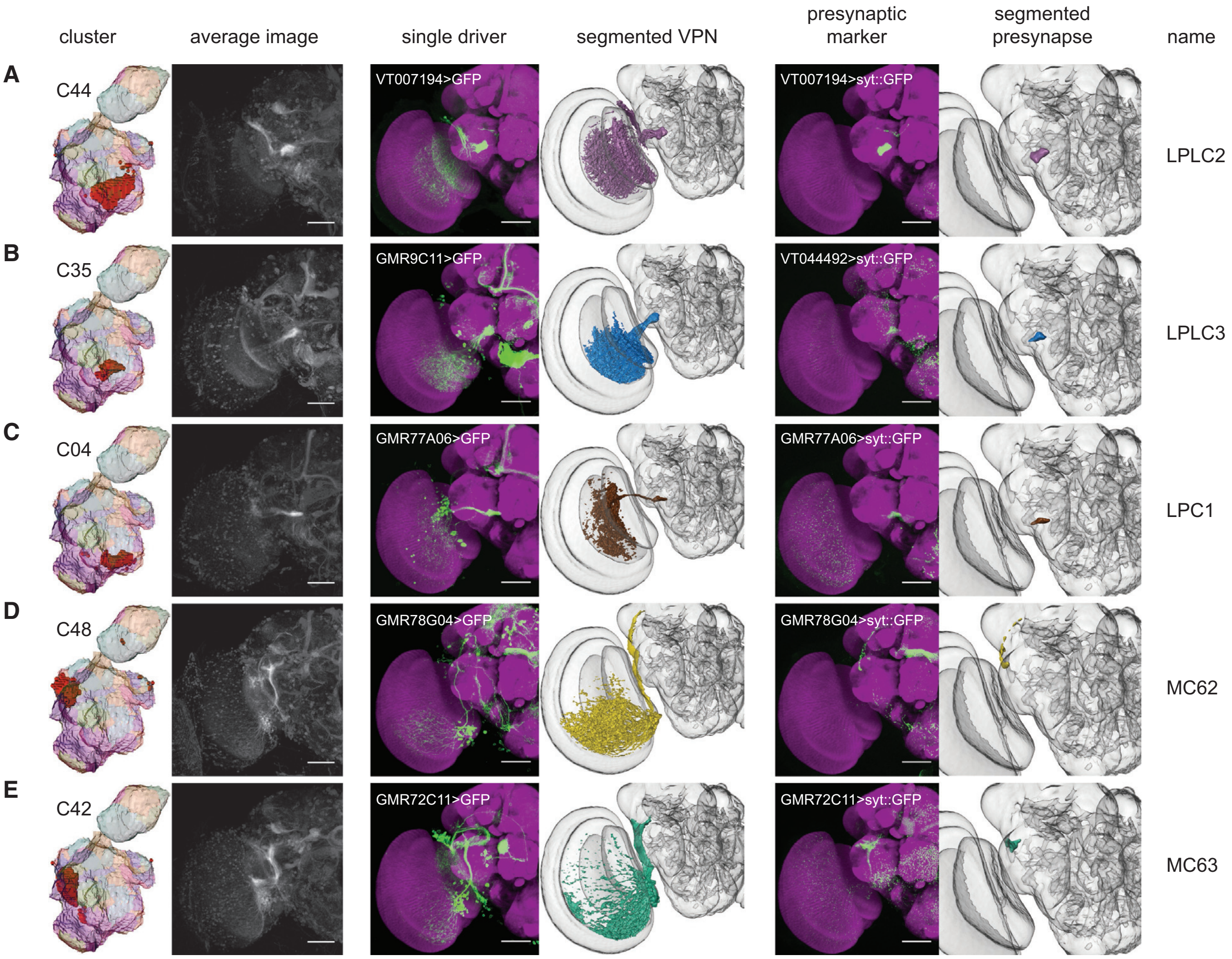

Figure 3. Automatic Segmentation Reveals Clusters that Correspond to Optic Glomeruli Associated with Newly Identified LPLC-, LPC-, and MC-type Visual Projection Neurons

Individual clusters, average images, selected driver lines, 3D segmentations of a particular VPN type, a presynaptic marker (UAS-synaptotagmin::GFP) expressed by a single driver, and 3D segmentation of the presynaptic region to define the optic glomerulus (A–E). Janelia FlyLight data for the oVLNP, run 1, 42,317 voxels, 3,462 driver lines, $k = 60$. Scale bars, 50 μm. See also Figures S2 and S3.

discussed above, the specific location and identity of structures within these regions remain incompletely described. Therefore, we used Braincode to identify putative functional units in this region (Figures 1D–1F, 2, 3, and 4).

Consistent with the idea that some of the automatically segmented clusters are optic glomeruli, we could identify a single, previously described VPN type projecting to many of these clusters (Figure 1E). In addition to creating an average image by combining driver lines expressing in the cluster, we selected individual driver lines that appeared to drive expression in a single VPN type projecting to this cluster. By comparing the morphology of the neurons selected this way with previous reports, particularly Otsuna and Ito [14], we could identify LC04, LC06, LC09, LC10, LC11, LC12, LC13, and LC14. To image the precise location of synaptic outputs of each of these VPN types, we expressed a presynaptic marker, synaptotagmin::GFP (syt::GFP) [53], using the selected driver lines. After registering these newly acquired confocal image z stacks to the templates of the Vienna or Janelia collections, we could then define the 3D location and extent of the VPN output—the VPN's associated optic glomerulus—by performing assisted 3D segmentations of the presynaptic regions. Initial inspection showed a substantial similarity between such manually validated optic glomeruli and automatically identified clusters, and below we quantify this correspondence.

When segmenting a large brain region into putative functional units, we might expect to find axon tracts in addition to nuclei or glomeruli. Indeed, the clustering results also included two apparent axon tracts through this region, the great commissure connecting the two contralateral lobulae including LC14 and the tract that includes the lamina tangential neuron type (Vienna dataset, $k = 60$, run 1, clusters 3 and 30, respectively).

In addition to clusters corresponding to output regions of previously identified neuron types, we found clusters that appear to be projection targets of VPNs that have not been previously described. These novel VPNs are eight lobula columnar types

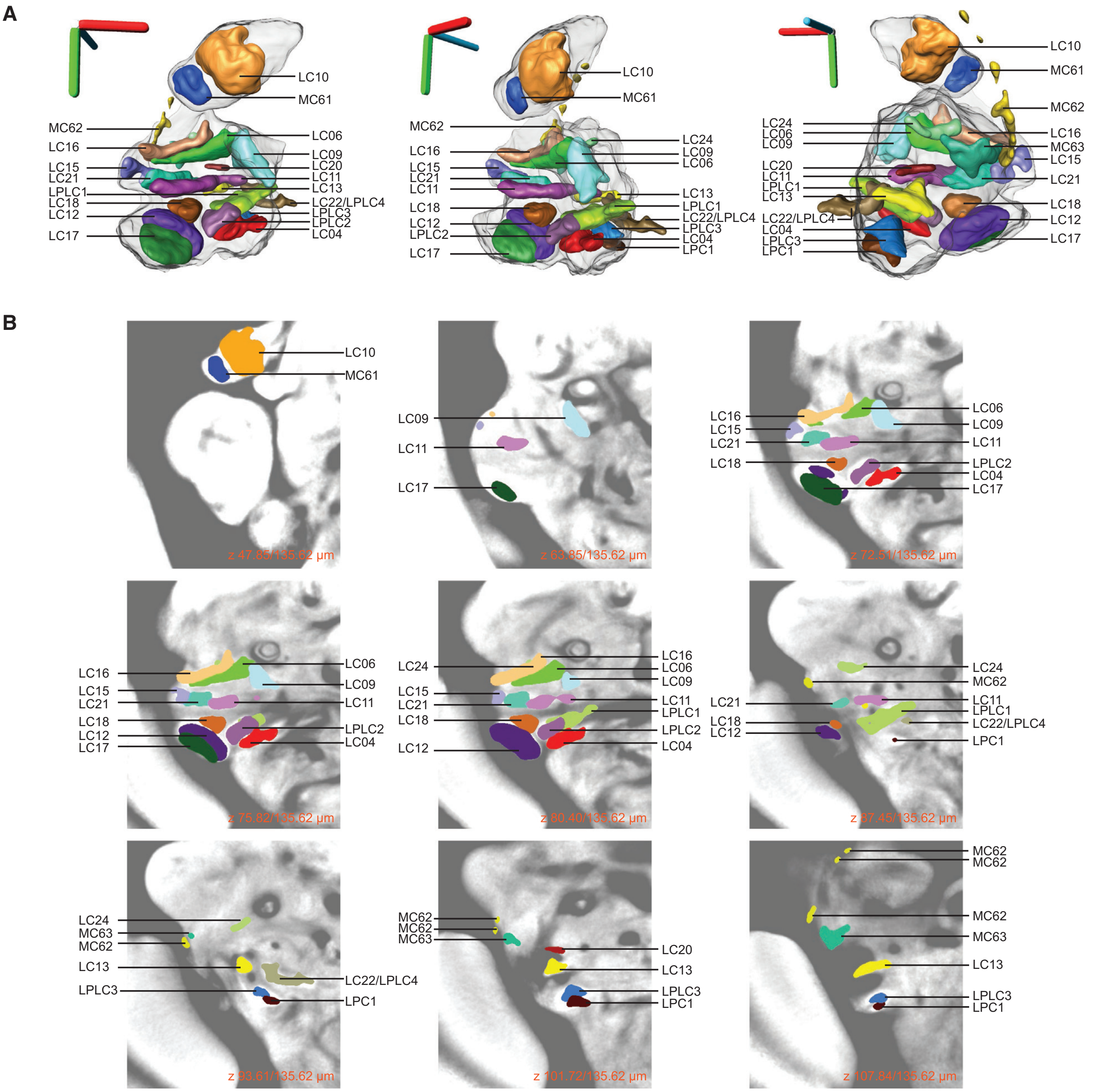

Figure 4. An Atlas of the Optic Glomeruli Defined by Manual Segmentation of Presynaptic Marker Expression Experiments

(A) Three-dimensional rendering of all identified optic glomeruli registered onto a 3D reference brain. Optic glomeruli were segmented from single-driver confocal images expressing a presynaptic marker (UAS-synaptotagmin::GFP). Scale bars, 40 μm in lateral (red), dorsal-ventral (green), and anterior-posterior (blue).

(B) Z stack showing the location of each optic glomerulus in a 2D view on the background of an average image of many individual nc82-stained brains.

See also Table S1 and Movie S1.

(Figure 2), three lobula plate-lobula columnar (LPLC) types, one lobula-plate columnar (LPC) type, and two medulla columnar (MC) types (Figure 3). Using the same presynaptic GFP expression approach as above, we saw substantial similarity between these manually validated optic glomeruli to the clustering result (Figures 2 and 3). For each cell type, we used the FlyCircuit database [23] to identify multiple example single-neuron morphologies (Table S1). We named these neuron types by continuing the sequence onward from the last published number for a particular class (i.e., LC15 is the first lobula columnar type we identified, whereas LC14 was previously reported).

We defined the precise 3D location of the optic glomeruli by segmenting the presynaptic marker signal from registered confocal image stacks of VPN lines. Quantification showed a high degree of colocalization between these manually validated optic glomeruli and voxels from specific clusters, and plotting these results showed that the Braincode method automatically produces segmentations with substantial similarity to those

derived from labor-intensive manual techniques (Figures S3A and S3B). This holds true across a second, entirely distinct dataset (Figures S3C and S3D).

We evaluated the completeness of the results in two ways. First, we clustered both datasets twice with $k = 60$ but different random number seeds and discovered in each run at least 23 of the 25 glomeruli or tracts associated with a particular VPN type (run 1 and run 2; Table S1). We expect subsequent repetitions to reveal few, if any, additional novel structures. Second, we noted that regions of high-intensity anti-Bruchpilot (nc82 antibody) staining, an indicator of synaptic contacts, coincide with optic glomeruli. In the brain regions investigated, we found glomeruli for all such high-intensity regions (Figure 4). We performed further exploration of the oVLNP clusters that did not obviously correspond to optic glomeruli. In most cases, ascribing an identity is difficult because relevant driver lines often have diffuse projection patterns and, even after viewing many individual examples, it is hard to discern underlying structure. In some cases, it seems that clusters form from expression patterns not in regions of dense synaptic contact in our analysis region. For example, we found a cluster (C14, Janelia run 1, $k = 60$) at the dorsal edge of the medial margin of the oVLNP, which contains cell bodies of ellipsoid body neurons, and another example (C06) containing a sub-esophogeal zone (SEZ) tract passing just within the margin of our analysis region. We did not perform clustering on the posterior slope, a region targeted by the lobula-plate tangential cells, and therefore, as expected, did not find any such clusters corresponding to these cells. Taking these results together, we conclude that the Braincode method can find a majority of structures in a particular region.

Little VPN Convergence to Single Optic Glomeruli

Of the 22 optic glomeruli we identified, only a single one was targeted by two VPN types. Apart from LC22 and LPLC4 projecting to the same glomerulus, we found no other instance of convergence of multiple VPN types to a single optic glomerulus. In some cases, however, two VPN types projected to a single cluster. For example, LC11 and LC21 both project to the region containing C07 (Figures 1E and 2F). Although there are some regions of presynaptic colocalization in the underlying signals in the registered images, there are also non-overlapping presynaptic localizations, and thus the data suggest that the glomeruli are at least partially distinct (Figure 4B). LC12 and LC17 are another similar pair, but the presynaptic localization is more distinct in this case (Figure 4B). Similarly, the presynaptic localizations of LC16 and LC24 both are within cluster C37, although in this case we think that a paucity of driver lines driving expression in LC24 likely precluded a separate cluster from being identified. In summary, with a single exception, we do not find evidence for multiple VPNs projecting to a single optic glomerulus and instead propose that, where we do see projection to the same cluster, this results from lumping within the clustering algorithm.

Although we cannot exclude the possibility that more optic glomeruli exist that are the targets of two or more VPN types, our data show that such cases are exceptional. Conversely, we found that each VPN type projects to a single glomerulus. Together, these two observations allow us to propose naming optic glomeruli according to the VPN type(s) that projects to them.

A Map of the Optic Glomeruli of *Drosophila*

We synthesized the novel findings of this automatic and manual characterization of this brain region with a movie showing segmented visual projection neurons and the presynaptic output regions associated with each of these VPNs (Movie S1) and created a figure describing the optic glomeruli as the targets of specific VPNs (Figure 4). Three-dimensional models of each VPN type and associated optic glomerulus are in the Supplemental Information. The optic glomeruli are generally dispersed in the oVLNP, separated by regions with reduced presynaptic signal, in contrast to the densely packed olfactory glomeruli. Glomeruli associated with LPC and LPLC neurons from the lobula plate project to a compact region in the medioposterior part of the central brain, whereas glomeruli of LC neurons from the lobula are dispersed throughout the oVLNP (Figure 5A). A few glomeruli are apposed, without clear buffer regions between them. LC12 and LC17 glomeruli are in close proximity without a clear boundary between them, and share the same cell-body region and have a similar projection path. This holds also for LC11 and LC21, as well as LPC1 and LPLC3. Another example of glomeruli in close proximity is LC06 and LC16, but these two neuron types have separate cell-body regions, and axons of these two neuron types enter the central brain from completely different sides.

We consistently found six subtrees in the hierarchical agglomerations from runs initialized with different random seeds, and asked whether this evidence of similar hierarchical relationships between enhancer expression patterns was reflected in the spatial organization of the VPNs or their glomeruli (Figures 5B and 5C). Indeed, glomeruli associated with these groups are always near each other, whereas cell-body location of the associated VPNs was not consistently a shared feature within such a group. Given that cell bodies do not migrate during development, this suggests that these hierarchical arrangements may reflect axon-targeting programs rather than neuroblast identity.

Is retinotopy, characteristic of the optic lobes, maintained in the projection to single optic glomeruli? Using MARCM [54] with LC04 and LC06 drivers, we found that presynaptic varicosities on axonal branches of single neurons are distributed across most of the glomerulus (Figure 5D), indicating that retinotopy within these glomeruli is unlikely. In contrast, the MC61 cell type showed localized termination within the lateral AOTU (Figure 5D). Examination of other identified neuron types (Table S1) using the FlyCircuit database [23] suggests that most columnar VPNs follow the example of LC04 and LC06, consistent with work on blow flies [16, 19].

DISCUSSION

We have demonstrated that applying a clustering algorithm to image data from genomic-scale enhancer libraries segments brain regions into smaller, putative functional units such as glomeruli and axon tracts. When applied to *Drosophila* data, automatically extracted clusters have a high correspondence with glomeruli and other neuropil subdivisions within the antennal lobes and central complex, suggesting the utility of the approach. We used this approach to inform a detailed investigation of the optic ventrolateral neuropil, a region where most outputs from the medulla and lobula neuropils within the optic

CellPress

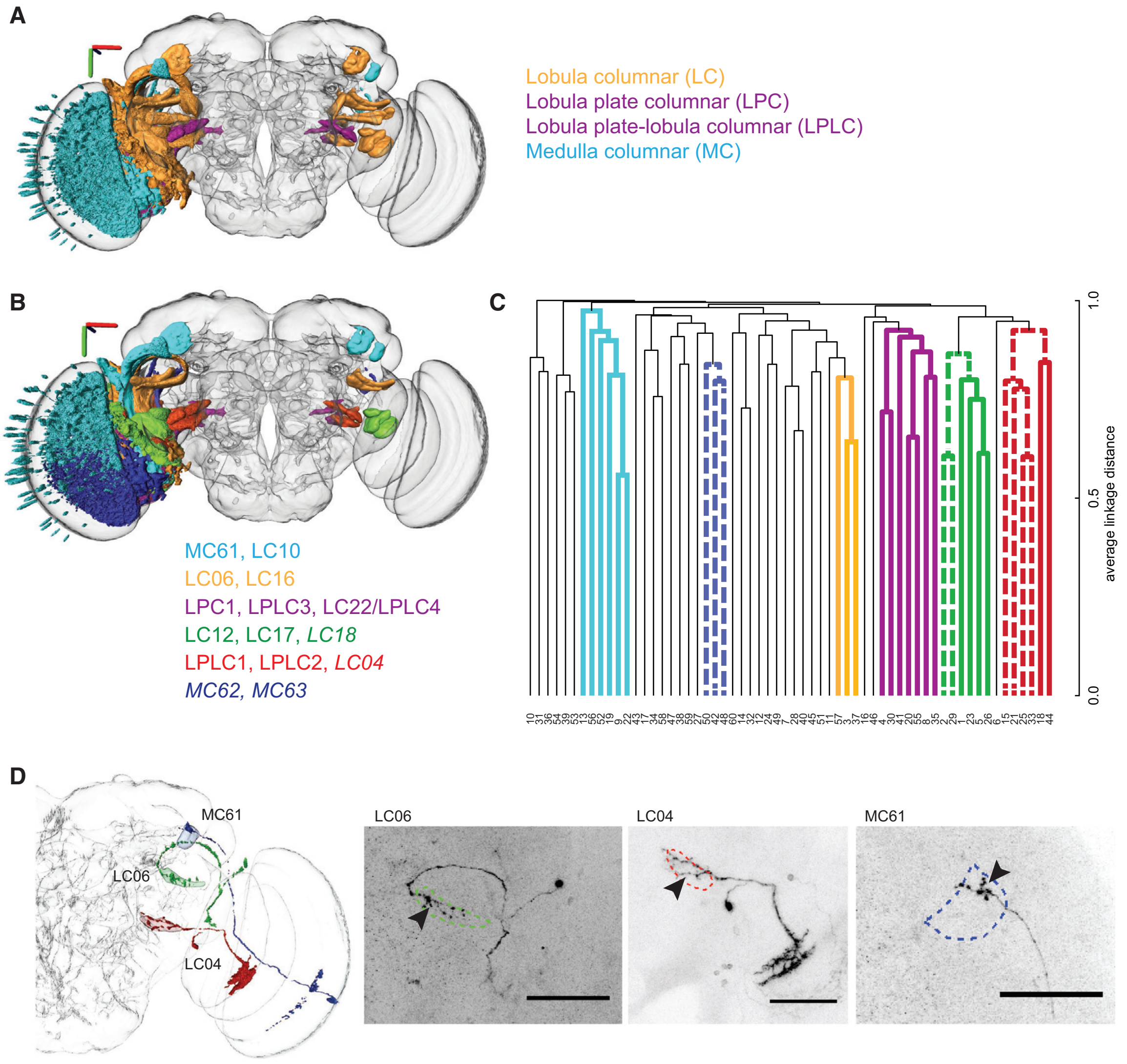

Figure 5. Distribution of Optic Glomeruli within the Lateral Protocerebrum and VPN Axons within Glomeruli

(A) VPNs exclusively from the lobula (orange; LC types) project to optic glomeruli distributed throughout the oVLNP, whereas those from the medulla (cyan; MC) and lobula plate (purple; LPLC and LPC) project to restricted areas of the oVLNP. Three-dimensional axis scale, 40 μm.

(B) Optic glomeruli that consistently group together across repeated clustering runs with different random initialization seeds. Groups correspond to subtrees in the dendrogram for three of four runs (italics) or four of four runs (non-italics). Three-dimensional axis scale, 40 μm.

(C) Dendrogram with consistent hierarchies highlighted. Bold, colored lines correspond to (B) and subtrees in the dendrogram for three of four runs (dashed lines) or four of four runs (non-dashed lines). Dendrogram from Janelia FlyLight data for the oVLNP, run 1, 42,317 voxels, 3,462 driver lines, $k = 60$.

(D) MARCM analysis shows presynaptic varicosities distributed throughout optic glomeruli in single axons of LC04 and LC06 but localized for MC61. Arrowheads denote the glomerulus region. Scale bars, 50 μm. (Genotype for LC06 and MC61: *yw, neoFRT19A/hsFLP, tubGAL80, neoFRT19A; UAS-mCD8::GFP/+; VT009855-GAL4/+*; genotype for LC04: *yw, neoFRT19A/hsFLP, tubGAL80, neoFRT19A; UAS-mCD8::GFP/+; VT046005-GAL4/+*.)

lobes reach the central brain. We identified several neuron types that, to the best of our knowledge, have not been previously described: eight lobula columnar neuron types, three lobula plate-lobula columnar types, one lobula-plate columnar type, and two medulla columnar types.

We found a nearly one-to-one projection of visual projection neurons to optic glomeruli. This is consistent with the idea that each optic glomerulus processes input from a single cell type and is therefore similar to the olfactory glomeruli in the sense that a dedicated glomerulus receives input from a single distinct input cell type [13]. Future work could investigate whether the optic and olfactory glomeruli are homologous in an evolutionary sense and whether the similarities extend to functional aspects and developmental mechanisms.

Recent computational neuroanatomical work has sought to use extensive collections of registered image stacks from stochastically labeled brains [23] to identify cell types [52], construct a mesoscale connectome of the fly brain [27], or find groups of morphologically similar neurons likely from the same neuroblast [55]. Given the complementary strengths of the respective approaches—resolution to the single-cell level with stochastic labeling, and candidate driver lines and molecular identity from

the present Braincode method—it may be productive to perform further analysis that combines these techniques.

The approach outlined here has several technical dependencies, which may represent limitations in some cases. First, any structure segmented automatically must have a physical scale at least comparable to, if not larger than, the error in registering multiple samples. Second, enough registered enhancer line images must be available to provide a signal sufficient for clustering. Third, underlying biological variability in developmental patterns must be sufficiently low. In addition to these technical dependencies, the use of an automatic classification algorithm does not solve the classic lumping-versus-splitting problem, and we propose using hierarchical agglomeration on finely split datasets to bypass such issues. Also, although we have shown that clustering often identifies regions with anatomical correlates such as a glomerulus, in other cases this may be less clear. In any case, the clusters identified result from patterns of expression in many driver lines, but it may be that only some driver lines are confined to the boundaries of a given cluster. In cases where the automatically extracted clusters do not clearly correspond to an anatomical structure, we propose that clustering may nonetheless be useful in reducing the complexity of thinking about a large brain region by dividing it into smaller elements.

Despite these potential limitations, the Braincode approach is not limited to *Drosophila*. Data are available from recent zebrafish enhancer-trap experiments [56, 57], and registering brains is also possible [58]. Together, these would enable an attempt to apply the Braincode technique. New developments, such as the use of site-specific integrase [59, 60], could be used to minimize expression-level variation due to effects of where a transgene integrates in the genome and improve efficiency and thus produce comparable datasets to those used here for *Drosophila*. Such an effort in zebrafish could be used to suggest driver lines corresponding to functional units identified in brain-wide activity-based experiments [61–64]. Similar datasets are being gathered in another fish species, medaka [65]. Variability of brain development in mammals may make the approach more challenging, or only operate on larger scales, in these species. Nevertheless, the ability to automatically segment brain regions into putative functional units could prove useful in unraveling structure-function relationships in a variety of species.

EXPERIMENTAL PROCEDURES

Drosophila Strains/Stocks

Flies were raised at 25°C under a 12-hr light-dark cycle on standard cornmeal food. GAL4 lines were from the Vienna Tiles collection (generated by the group of B.J. Dickson with help from A. Stark, personal communication; see also Kvon et al. [2]) and Janelia GAL4 library [3, 66] and were obtained from the Vienna *Drosophila* RNAi Center or Bloomington *Drosophila* Stock Center, respectively. *UAS-mCD8::GFP* stock is from B.J. Dickson and *UAS-DenMark::mCherry, UAS-synaptotagmin::GFP* is from Bloomington 33064. For MARCM analysis, we used *yw, neoFRT19A; If/CyO; Sb/Tm3Ser* and *hsFLP, tubGAL80, neoFRT19A; UAS-mCD8::GFP/CyO; Sb/Tm3Ser*. So that stochastic single-cell labeling could be obtained, flies were heat shocked for 2 hr at 37°C during larval stages and then normally developed to adults at 25°C.

Sample Preparation and Imaging

Fly dissection and staining were performed as previously described [67] using 3- to 5-day-old adult flies. In brief, brains were dissected in PBS, fixed in 4% paraformaldehyde in PBS with 0.1% Triton X-100, and subsequently blocked in 10% normal goat serum (Gibco Life Technologies). Brains were incubated in primary and secondary antibodies for a minimum of 20 hr at 4°C and washed in PBS with 0.3% Triton X-100. Fly brains were mounted in Vectashield (Vector Laboratories). We used the following primary antibodies: rabbit polyclonal anti-GFP (1:5,000, TP401; Torrey Pines), mouse monoclonal anti-Bruchpilot (1:20, nc82; Developmental Studies Hybridoma Bank), and chicken polyclonal anti-GFP (1:10,000, ab13970; Abcam) We used the following secondary antibodies: Alexa Fluor 488, 568, and 633 antibodies (1:500–1:1,000; Invitrogen Life Technologies).

Images were acquired using point scanning confocal microscope LSM 780 or LSM 700 (Zeiss) equipped with 25×/0.8 plan-apochromat multi-immersion or 20×/0.8 plan-apochromat dry objectives, respectively. For avoidance of channel cross-talk, confocal z stacks were recorded in the multi-track (LSM 700) or online fingerprinting mode (LSM 780).

Registration, Assisted Segmentation, and 3D Rendering

For both datasets an intensity-based non-linear warping method was used. For the Vienna Tiles dataset, we used the approach described in [67] and, for the Janelia dataset, brains were registered according to [68]. Fiji (ImageJ; NIH) and Amira (4.1.2; Mercury Computer Systems) software were used for image processing and analysis. Amira label field function was used to segment optic glomeruli, projections, and neuron types from registered images. Surface files of segmented structures were generated using constrained smoothing for full neuron segmentations and unconstrained smoothing for optic glomeruli. We additionally used BrainGazer visualization software [69]. In all 3D figures, we included a 3D axis scale in which red specifies the lateral axis with positive toward the animal's left side, green specifies the dorsal-ventral axis with positive toward ventral, and blue specifies the anterior-posterior with positive toward posterior. Due to the use of a perspective projection in these figures, the size of the 3D axis scale is only approximate.

k-Medoids Clustering

As input, we took confocal image stacks from the G. Rubin laboratory Janelia FlyLight collection [1, 3] and from the B.J. Dickson laboratory Vienna Tiles collection (B.J. Dickson, personal communication). In total, we used data from 3,462 Janelia FlyLight and 6,022 Vienna Tiles GAL4 driver lines crossed with *UAS-mCD8::GFP*. Each dataset came registered to a dataset-specific template brain with registration error estimated to be 2–3 μm [67, 68]. We clustered these data in two major steps. First, within each analyzed brain region, we found *k* clusters (typically 60) using a conventional clustering algorithm, *k*-medoids. Second, we agglomerated these *k* original "singleton" clusters into a hierarchical structure in which closely related groups are merged successively. See the Supplemental Experimental Procedures for details.

Nomenclature

Existing nomenclature was used for previously identified neuron types when an unambiguous match was possible. Lobula columnar neurons were first systematically described in *Drosophila* in [4], which called these "Lcn" types and included Lcn1, Lcn2, Lcn4, Lcn5, Lcn6, Lcn7, and Lcn8 (Lcn3 was skipped). Later, these were named "LC" neurons, only unambiguous identities were maintained, and new numbers were given by [14]. In Otsuna and Ito's work [14], only Lcn4 and Lcn6 could be identified and became LC4 and LC6. (Due to uncertain identification, Lcn1, Lcn2, Lcn5, Lcn7, and Lcn8 have no LC counterpart.) In addition to LC4 and LC6, Otsuna and Ito identified LC9, LC10, LC11, LC12, LC13, and LC14. Naming of non-described types was based on the style of Otsuna and Ito and done in coordination with A. Nern and G. Rubin. Neuropils are referred to using the terminology of the Insect Brain Name Working Group [70].

SUPPLEMENTAL INFORMATION

Supplemental Information includes Supplemental Experimental Procedures, three figures, one table, one movie, and one dataset and can be found with this article online at http://dx.doi.org/10.1016/j.cub.2016.05.052.

AUTHOR CONTRIBUTIONS

K.P., L.T., F.S., and A.D.S. designed the research. K.P. performed the neuroanatomical experiments. K.P. and L.T. registered image data and identified

cell types and brain regions. K.P., L.T., F.S., S.V., and A.D.S. performed the data analysis. K.P., L.T., F.S., S.V., G.S.X.E.J., K.B., and A.D.S. interpreted the results and wrote the manuscript.

ACKNOWLEDGMENTS

We thank Barry Dickson for access to the Vienna Tiles library and for comments on the manuscript. We discussed with Aljoscha Nern and Gerry Rubin a common nomenclature for the VPNs. We thank the Janelia FlyLight team and the Dickson laboratory for providing the datasets. The IMP/IMBA Bio-optics core facility provided extensive microscopy support. Arnim Jenett provided a 3D atlas of brain regions. Veit Grabe and Silke Sachse provided a 3D atlas of the antennal lobes. We thank Gaby Maimon, David Hain, and the anonymous reviewers for helpful comments on the manuscript. This work was supported by ERC starting grant 281884 "FlyVisualCircuits" (to A.D.S.), FFG Headquarter grant 834223 to the IMP and VRVis, and IMP core funding.

Received: December 23, 2015
Revised: April 14, 2016
Accepted: May 20, 2016
Published: July 14, 2016

REFERENCES

1. Jenett, A., Rubin, G.M., Ngo, T.-T.B., Shepherd, D., Murphy, C., Dionne, H., Pfeiffer, B.D., Cavallaro, A., Hall, D., Jeter, J., et al. (2012). A GAL4-driver line resource for *Drosophila* neurobiology. Cell Rep. *2*, 991–1001.
2. Kvon, E.Z., Kazmar, T., Stampfel, G., Yáñez-Cuna, J.O., Pagani, M., Schernhuber, K., Dickson, B.J., and Stark, A. (2014). Genome-scale functional characterization of *Drosophila* developmental enhancers in vivo. Nature *512*, 91–95.
3. Pfeiffer, B.D., Jenett, A., Hammonds, A.S., Ngo, T.-T.B., Misra, S., Murphy, C., Scully, A., Carlson, J.W., Wan, K.H., Laverty, T.R., et al. (2008). Tools for neuroanatomy and neurogenetics in *Drosophila*. Proc. Natl. Acad. Sci. USA *105*, 9715–9720.
4. Fischbach, K.-F., and Dittrich, A.P.M. (1989). The optic lobe of *Drosophila melanogaster*. I. A Golgi analysis of wild-type structure. Cell Tissue Res. *258*, 441–475.
5. Fischbach, K.-F., and Lyly-Hünerberg, I. (1983). Genetic dissection of the anterior optic tract of *Drosophila melanogaster*. Cell Tissue Res. *231*, 551–563.
6. Nern, A., Pfeiffer, B.D., and Rubin, G.M. (2015). Optimized tools for multicolor stochastic labeling reveal diverse stereotyped cell arrangements in the fly visual system. Proc. Natl. Acad. Sci. USA *112*, E2967–E2976.
7. Raghu, S.V., and Borst, A. (2011). Candidate glutamatergic neurons in the visual system of *Drosophila*. PLoS ONE *6*, e19472.
8. Raghu, S.V., Joesch, M., Borst, A., and Reiff, D.F. (2007). Synaptic organization of lobula plate tangential cells in *Drosophila*: gamma-aminobutyric acid receptors and chemical release sites. J. Comp. Neurol. *502*, 598–610.
9. Raghu, S.V., Joesch, M., Sigrist, S.J., Borst, A., and Reiff, D.F. (2009). Synaptic organization of lobula plate tangential cells in *Drosophila*: Dalpha7 cholinergic receptors. J. Neurogenet. *23*, 200–209.
10. Varija Raghu, S., Reiff, D.F., and Borst, A. (2011). Neurons with cholinergic phenotype in the visual system of *Drosophila*. J. Comp. Neurol. *519*, 162–176.
11. Aptekar, J.W., Keleş, M.F., Lu, P.M., Zolotova, N.M., and Frye, M.A. (2015). Neurons forming optic glomeruli compute figure-ground discriminations in *Drosophila*. J. Neurosci. *35*, 7587–7599.
12. Ito, M., Masuda, N., Shinomiya, K., Endo, K., and Ito, K. (2013). Systematic analysis of neural projections reveals clonal composition of the *Drosophila* brain. Curr. Biol. *23*, 644–655.
13. Mu, L., Ito, K., Bacon, J.P., and Strausfeld, N.J. (2012). Optic glomeruli and their inputs in *Drosophila* share an organizational ground pattern with the antennal lobes. J. Neurosci. *32*, 6061–6071.
14. Otsuna, H., and Ito, K. (2006). Systematic analysis of the visual projection neurons of *Drosophila melanogaster*. I. Lobula-specific pathways. J. Comp. Neurol. *497*, 928–958.
15. Otsuna, H., Shinomiya, K., and Ito, K. (2014). Parallel neural pathways in higher visual centers of the *Drosophila* brain that mediate wavelength-specific behavior. Front. Neural Circuits *8*, 8.
16. Okamura, J.-Y., and Strausfeld, N.J. (2007). Visual system of calliphorid flies: motion- and orientation-sensitive visual interneurons supplying dorsal optic glomeruli. J. Comp. Neurol. *500*, 189–208.
17. Strausfeld, N.J., and Bacon, J.P. (1983). Multimodal convergence in the central nervous system of dipterous insects. In Fortschritte der Zoologie: Multimodal Convergence in Sensory Systems, *Vol. 28*, E. Horn, ed. (Gustav Fischer Verlag), pp. 47–76.
18. Strausfeld, N.J., and Lee, J.-K. (1991). Neuronal basis for parallel visual processing in the fly. Vis. Neurosci. *7*, 13–33.
19. Strausfeld, N.J., and Okamura, J.-Y. (2007). Visual system of calliphorid flies: organization of optic glomeruli and their lobula complex efferents. J. Comp. Neurol. *500*, 166–188.
20. Strausfeld, N.J., Sinakevitch, I., and Okamura, J.-Y. (2007). Organization of local interneurons in optic glomeruli of the dipterous visual system and comparisons with the antennal lobes. Dev. Neurobiol. *67*, 1267–1288.
21. Burkhardt, D., and de la Motte, I. (1983). How stalk-eyed flies eye stalk-eyed flies: observations and measurements of the eyes of *Cyrtodiopsis whitei* (Diopsidae, Diptera). J. Comp. Physiol. A Neuroethol. Sens. Neural Behav. Physiol. *151*, 407–421.
22. Alkemade, A., Keuken, M.C., and Forstmann, B.U. (2013). A perspective on terra incognita: uncovering the neuroanatomy of the human subcortex. Front. Neuroanat. *7*, 40.
23. Chiang, A.-S., Lin, C.-Y., Chuang, C.-C., Chang, H.-M., Hsieh, C.-H., Yeh, C.-W., Shih, C.-T., Wu, J.-J., Wang, G.-T., Chen, Y.-C., et al. (2011). Three-dimensional reconstruction of brain-wide wiring networks in *Drosophila* at single-cell resolution. Curr. Biol. *21*, 1–11.
24. Hadjieconomou, D., Rotkopf, S., Alexandre, C., Bell, D.M., Dickson, B.J., and Salecker, I. (2011). Flybow: genetic multicolor cell labeling for neural circuit analysis in *Drosophila melanogaster*. Nat. Methods *8*, 260–266.
25. Hampel, S., Chung, P., McKellar, C.E., Hall, D., Looger, L.L., and Simpson, J.H. (2011). *Drosophila* Brainbow: a recombinase-based fluorescence labeling technique to subdivide neural expression patterns. Nat. Methods *8*, 253–259.
26. Livet, J., Weissman, T.A., Kang, H., Draft, R.W., Lu, J., Bennis, R.A., Sanes, J.R., and Lichtman, J.W. (2007). Transgenic strategies for combinatorial expression of fluorescent proteins in the nervous system. Nature *450*, 56–62.
27. Shih, C.-T., Sporns, O., Yuan, S.-L., Su, T.-S., Lin, Y.-J., Chuang, C.-C., Wang, T.-Y., Lo, C.-C., Greenspan, R.J., and Chiang, A.-S. (2015). Connectomics-based analysis of information flow in the *Drosophila* brain. Curr. Biol. *25*, 1249–1258.
28. Yu, H.H., Awasaki, T., Schroeder, M.D.D., Long, F., Yang, J.S.S., He, Y., Ding, P., Kao, J.C., Wu, G.Y.-Y., Peng, H., et al. (2013). Clonal development and organization of the adult *Drosophila* central brain. Curr. Biol. *23*, 633–643.
29. Cardona, A., Saalfeld, S., Preibisch, S., Schmid, B., Cheng, A., Pulokas, J., Tomancak, P., and Hartenstein, V. (2010). An integrated micro- and macroarchitectural analysis of the *Drosophila* brain by computer-assisted serial section electron microscopy. PLoS Biol. *8*, 17.
30. Helmstaedter, M., Briggman, K.L., Turaga, S.C., Jain, V., Seung, H.S., and Denk, W. (2013). Connectomic reconstruction of the inner plexiform layer in the mouse retina. Nature *500*, 168–174.
31. Takemura, S.Y., Bharioke, A., Lu, Z., Nern, A., Vitaladevuni, S., Rivlin, P.K., Katz, W.T., Olbris, D.J., Plaza, S.M., Winston, P., et al. (2013). A visual motion detection circuit suggested by *Drosophila* connectomics. Nature *500*, 175–181.

32. White, J.G., Southgate, E., Thomson, J.N., and Brenner, S. (1986). The structure of the nervous system of the nematode *Caenorhabditis elegans* (the mind of a worm). Philos. Trans. R. Soc. B Biol. Sci. *314*, 1–340.

33. Meinertzhagen, I.A., Takemura, S.y., Lu, Z., Huang, S., Gao, S., Ting, C.Y., Lee, C.H., Ting, C.-Y., and Lee, C.-H. (2009). From form to function: the ways to know a neuron. J. Neurogenet. *23*, 68–77.

34. Fakhry, A., and Ji, S. (2015). High-resolution prediction of mouse brain connectivity using gene expression patterns. Methods *73*, 71–78.

35. Lein, E.S., Hawrylycz, M.J., Ao, N., Ayres, M., Bensinger, A., Bernard, A., Boe, A.F., Boguski, M.S., Brockway, K.S., Byrnes, E.J., et al. (2007). Genome-wide atlas of gene expression in the adult mouse brain. Nature *445*, 168–176.

36. Ng, L., Bernard, A., Lau, C., Overly, C.C., Dong, H.-W., Kuan, C., Pathak, S., Sunkin, S.M., Dang, C., Bohland, J.W., et al. (2009). An anatomic gene expression atlas of the adult mouse brain. Nat. Neurosci. *12*, 356–362.

37. Thompson, C.L., Ng, L., Menon, V., Martinez, S., Lee, C.-K., Glattfelder, K., Sunkin, S.M., Henry, A., Lau, C., Dang, C., et al. (2014). A high-resolution spatiotemporal atlas of gene expression of the developing mouse brain. Neuron *83*, 309–323.

38. Goel, P., Kuceyeski, A., LoCastro, E., and Raj, A. (2014). Spatial patterns of genome-wide expression profiles reflect anatomic and fiber connectivity architecture of healthy human brain. Hum. Brain Mapp. *35*, 4204–4218.

39. Hawrylycz, M.J., Lein, E.S., Guillozet-Bongaarts, A.L., Shen, E.H., Ng, L., Miller, J.A., van de Lagemaat, L.N., Smith, K.A., Ebbert, A., Riley, Z.L., et al. (2012). An anatomically comprehensive atlas of the adult human brain transcriptome. Nature *489*, 391–399.

40. Mahfouz, A., van de Giessen, M., van der Maaten, L., Huisman, S., Reinders, M., Hawrylycz, M.J., and Lelieveldt, B.P.F. (2015). Visualizing the spatial gene expression organization in the brain through non-linear similarity embeddings. Methods *73*, 79–89.

41. Myers, E.M., Bartlett, C.W., Machiraju, R., and Bohland, J.W. (2015). An integrative analysis of regional gene expression profiles in the human brain. Methods *73*, 54–70.

42. Couto, A., Alenius, M., and Dickson, B.J. (2005). Molecular, anatomical, and functional organization of the *Drosophila* olfactory system. Curr. Biol. *15*, 1535–1547.

43. Grabe, V., Strutz, A., Baschwitz, A., Hansson, B.S., and Sachse, S. (2015). Digital in vivo 3D atlas of the antennal lobe of *Drosophila melanogaster*. J. Comp. Neurol. *523*, 530–544.

44. Vosshall, L.B., Wong, A.M., and Axel, R. (2000). An olfactory sensory map in the fly brain. Cell *102*, 147–159.

45. Bausenwein, B., Wolf, R., and Heisenberg, M. (1986). Genetic dissection of optomotor behavior in *Drosophila melanogaster*. Studies on wild-type and the mutant optomotor-blindH31. J. Neurogenet. *3*, 87–109.

46. Hanesch, U., Fischbach, K.-F., and Heisenberg, M. (1989). Neuronal architecture of the central complex in *Drosophila melanogaster*. Cell Tissue Res. *257*, 343–366.

47. Lin, C.-Y., Chuang, C.-C., Hua, T.-E., Chen, C.-C., Dickson, B.J., Greenspan, R.J., and Chiang, A.-S. (2013). A comprehensive wiring diagram of the protocerebral bridge for visual information processing in the *Drosophila* brain. Cell Rep. *3*, 1739–1753.

48. Strauss, R., and Heisenberg, M. (1993). A higher control center of locomotor behavior in the *Drosophila* brain. J. Neurosci. *13*, 1852–1861.

49. Wolff, T., Iyer, N.A., and Rubin, G.M. (2015). Neuroarchitecture and neuroanatomy of the *Drosophila* central complex: a GAL4-based dissection of protocerebral bridge neurons and circuits. J. Comp. Neurol. *523*, 997–1037.

50. Mota, T., Yamagata, N., Giurfa, M., Gronenberg, W., and Sandoz, J.-C. (2011). Neural organization and visual processing in the anterior optic tubercle of the honeybee brain. J. Neurosci. *31*, 11443–11456.

51. Homberg, U., Hofer, S., Pfeiffer, K., and Gebhardt, S. (2003). Organization and neural connections of the anterior optic tubercle in the brain of the locust, *Schistocerca gregaria*. J. Comp. Neurol. *462*, 415–430.

52. Costa, M., Manton, J.D., Ostrovsky, A.D., Prohaska, S., and Jefferis, G.S.X.E. (2016). NBLAST: rapid, sensitive comparison of neuronal structure and construction of neuron family databases. Neuron. Published online June 30, 2016. http://dx.doi.org/10.1016/j.neuron.2016.06.012.

53. Zhang, Y.Q., Rodesch, C.K., and Broadie, K. (2002). Living synaptic vesicle marker: synaptotagmin-GFP. Genesis *34*, 142–145.

54. Lee, T., and Luo, L. (1999). Mosaic analysis with a repressible cell marker for studies of gene function in neuronal morphogenesis. Neuron *22*, 451–461.

55. Masse, N.Y., Cachero, S., Ostrovsky, A.D., and Jefferis, G.S.X.E. (2012). A mutual information approach to automate identification of neuronal clusters in *Drosophila* brain images. Front. Neuroinform. *6*, 21.

56. Kawakami, K., Abe, G., Asada, T., Asakawa, K., Fukuda, R., Ito, A., Lal, P., Mouri, N., Muto, A., Suster, M.L., et al. (2010). zTrap: zebrafish gene trap and enhancer trap database. BMC Dev. Biol. *10*, 105.

57. Kondrychyn, I., Teh, C., Garcia-Lecea, M., Guan, Y., Kang, A., and Korzh, V. (2011). Zebrafish enhancer TRAP transgenic line database ZETRAP 2.0. Zebrafish *8*, 181–182.

58. Ronneberger, O., Liu, K., Rath, M., Rueβ, D., Mueller, T., Skibbe, H., Drayer, B., Schmidt, T., Filippi, A., Nitschke, R., et al. (2012). ViBE-Z: a framework for 3D virtual colocalization analysis in zebrafish larval brains. Nat. Methods *9*, 735–742.

59. Lister, J.A. (2011). Use of phage φC31 integrase as a tool for zebrafish genome manipulation. Methods Cell Biol. *104*, 195–208.

60. Mosimann, C., Puller, A.-C., Lawson, K.L., Tschopp, P., Amsterdam, A., and Zon, L.I. (2013). Site-directed zebrafish transgenesis into single landing sites with the phiC31 integrase system. Dev. Dyn. *242*, 949–963.

61. Ahrens, M.B., Li, J.M., Orger, M.B., Robson, D.N., Schier, A.F., Engert, F., and Portugues, R. (2012). Brain-wide neuronal dynamics during motor adaptation in zebrafish. Nature *485*, 471–477.

62. Kubo, F., Hablitzel, B., Dal Maschio, M., Driever, W., Baier, H., and Arrenberg, A.B. (2014). Functional architecture of an optic flow-responsive area that drives horizontal eye movements in zebrafish. Neuron *81*, 1344–1359.

63. Portugues, R., Feierstein, C.E., Engert, F., and Orger, M.B. (2014). Whole-brain activity maps reveal stereotyped, distributed networks for visuomotor behavior. Neuron *81*, 1328–1343.

64. Randlett, O., Wee, C.L., Naumann, E.A., Nnaemeka, O., Schoppik, D., Fitzgerald, J.E., Portugues, R., Lacoste, A.M.B., Riegler, C., Engert, F., and Schier, A.F. (2015). Whole-brain activity mapping onto a zebrafish brain atlas. Nat. Methods *12*, 1039–1046.

65. Alonso-Barba, J.I., Rahman, R.-U., Wittbrodt, J., and Mateo, J.L. (2016). MEPD: medaka expression pattern database, genes and more. Nucleic Acids Res. *44*, D819–D821.

66. Pfeiffer, B.D., Ngo, T.-T.B., Hibbard, K.L., Murphy, C., Jenett, A., Truman, J.W., and Rubin, G.M. (2010). Refinement of tools for targeted gene expression in *Drosophila*. Genetics *186*, 735–755.

67. Yu, J.Y., Kanai, M.I., Demir, E., Jefferis, G.S.X.E., and Dickson, B.J. (2010). Cellular organization of the neural circuit that drives *Drosophila* courtship behavior. Curr. Biol. *20*, 1602–1614.

68. Cachero, S., Ostrovsky, A.D., Yu, J.Y., Dickson, B.J., and Jefferis, G.S.X.E. (2010). Sexual dimorphism in the fly brain. Curr. Biol. *20*, 1589–1601.

69. Bruckner, S., Soltészová, V., Gröller, M.E., Hladůvka, J., Bühler, K., Yu, J.Y., and Dickson, B.J. (2009). BrainGazer—visual queries for neurobiology research. IEEE Trans. Vis. Comput. Graph. *15*, 1497–1504.

70. Ito, K., Shinomiya, K., Ito, M., Armstrong, J.D., Boyan, G., Hartenstein, V., Harzsch, S., Heisenberg, M., Homberg, U., Jenett, A., et al.; Insect Brain Name Working Group (2014). A systematic nomenclature for the insect brain. Neuron *81*, 755–765.

Current Biology, Volume *26*

Supplemental Information

Automatic Segmentation of *Drosophila* Neural Compartments Using GAL4 Expression Data Reveals Novel Visual Pathways

Karin Panser, Laszlo Tirian, Florian Schulze, Santiago Villalba, Gregory S.X.E. Jefferis, Katja Bühler, and Andrew D. Straw

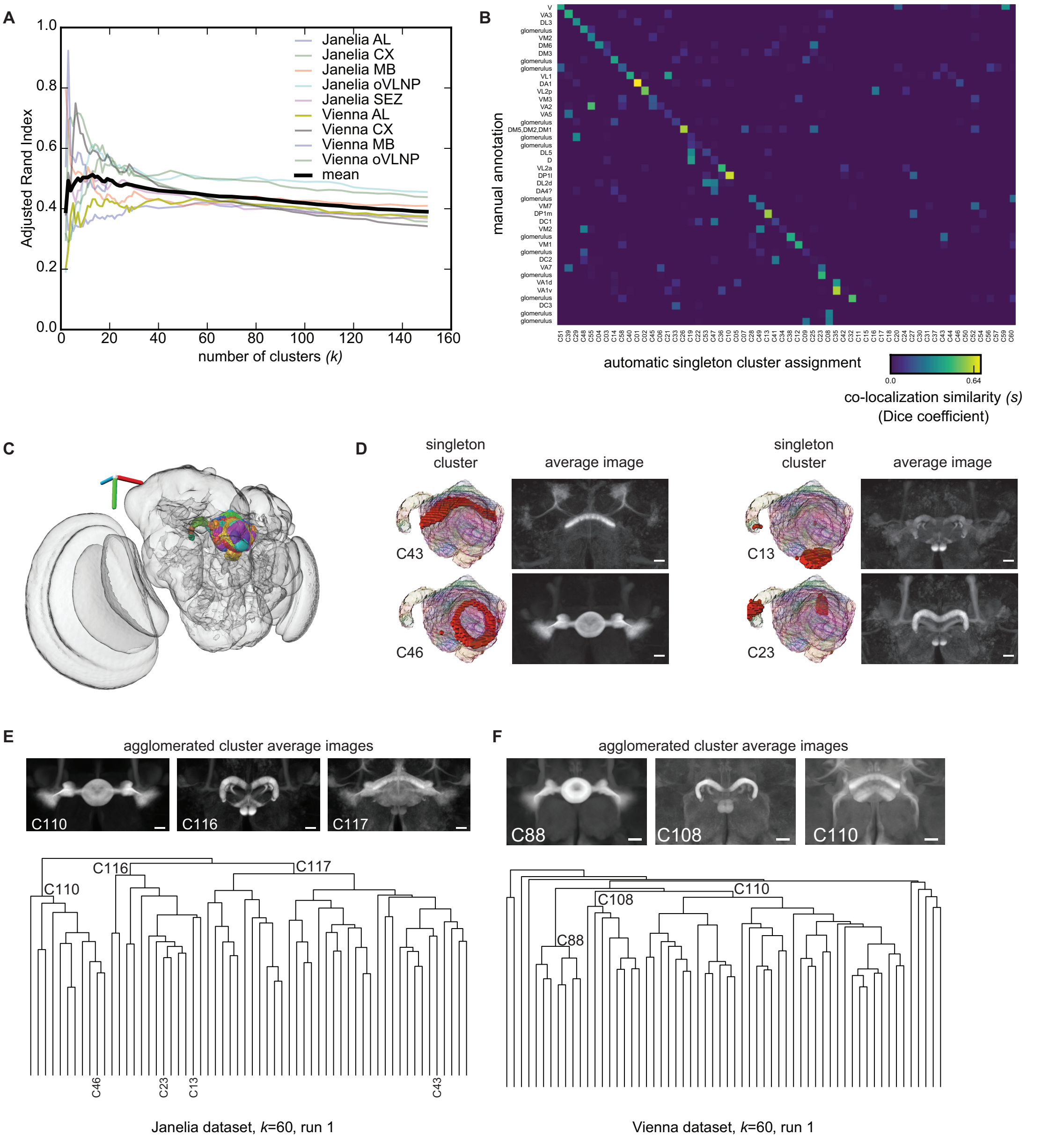

Figure S1. (data related to Figure 1): Evaluation of *k*-medoids clustering for automatically segmenting brain regions into anatomical structures.

(A) Repeatability scores across multiple runs of the *k*-medoids algorithm. The adjusted Rand index, a measure of repeatability, was calculated based on 10 repeated runs of the *k*-medoids algorithm for both datasets and several brain regions.

(B) Colocalization similarity (measured as Dice coefficient *s* on the set of voxels in the manually annotated region and the set in the clustering result) between the Janelia FlyLight dataset and manual assignments using the same 3D template brain. Manual assignments were based on a manually segmented neuropil image. Glomeruli that could not be unambiguously identified were labeled “glomerulus”. (Janelia FlyLight data for the right antennal lobe region, run 1, 6502 voxels, 3462 driver lines, *k* equal 60.)

(C) Automatic segmentation of central complex (CX). 3D axes scale 30 µm.

(D) Individual singleton clusters (left) and average image of strongly expressing driver lines in each cluster with broad driver lines removed (right). Scale bars 20 µm.

(E) Average images from agglomerated clusters (top) and dendrogram of agglomerated hierarchy. Scale bars 20 µm.

(F) As in E, but from the Vienna dataset, *k*=60. Scale bars 20 µm.

Panels C-E: Janelia FlyLight data for CX, run 1, 27598 voxels, 3462 driver lines, *k*=60.

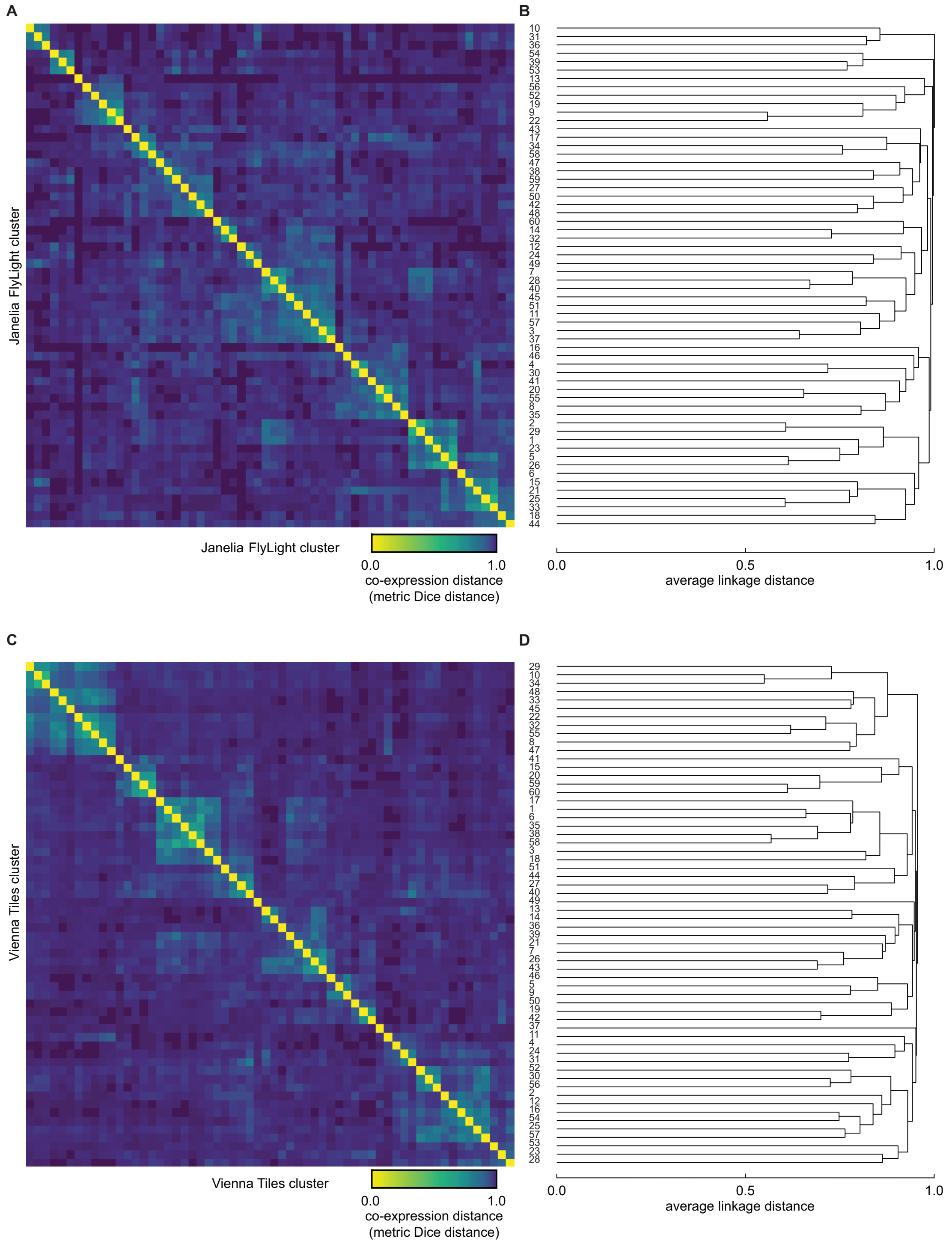

Figure S2. (data related to Figures 2-3): Clustering quality for oVLNP in both datasets.

(A) Quantification of similarity between clusters as measured by voxel-to-voxel co-expression distance ($\sqrt{1-s}$, where s is the Dice coefficient between the two sets of enhancer expression) for each medoid of every cluster of run 1 in the oVLNP region using the Janelia dataset.

(B) Dendrogram of agglomerative hierarchical clustering using average linkage showing a representation of co-expression distance between medoids in the oVLNP region of the Janelia dataset.

(C) Quantification of similarity between clusters as measured by voxel-to-voxel co-expression distance for each medoid of every cluster in the oVLNP region of run 1 the Vienna dataset.

(D) Dendrogram as in B using the Vienna dataset.

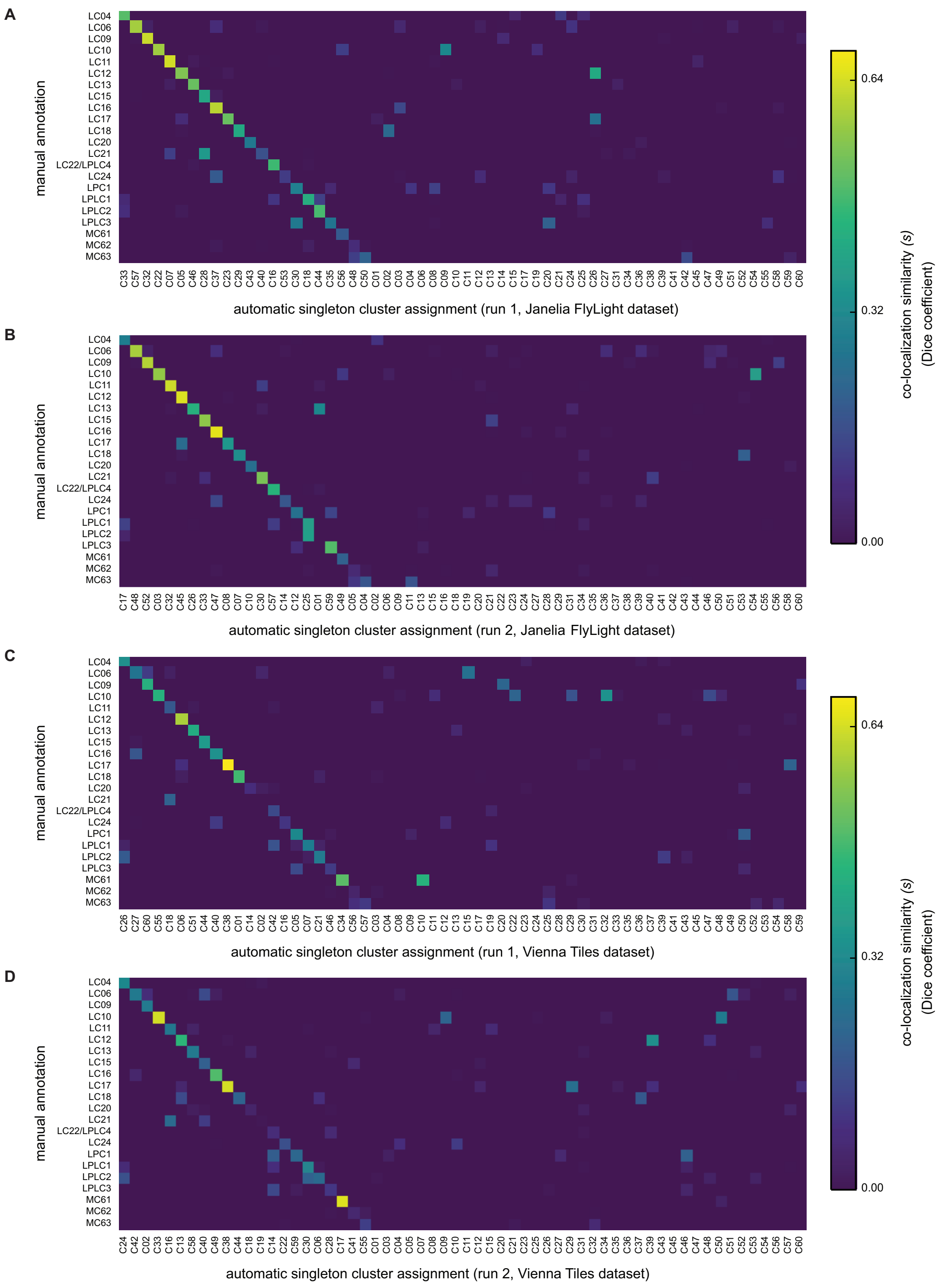

Figure S3. (data related to Figures 2-3): Automatically assigned oVLNP singleton clusters colocalize with manually segmented optic glomeruli, repeated clustering of the same dataset gives similar results, and clustering of different datasets gives similar results.

(A-B) Colocalization similarity (measured as Dice coefficient *s* on the set of voxels in the manually annotated region and the set in the clustering result) between the Janelia FlyLight dataset and manual assignments using the same 3D template brain. (Janelia FlyLight data for oVLNP, 42317 voxels, 3462 driver lines, *k* equal 60.)

(C-D) Colocalization similarity between the Vienna Tiles dataset and manual assignments using the same 3D template brain. (Vienna Tiles data for oVLNP, 13458 voxels, 6022 driver lines, *k* equal 60.)

VPN type	Synonyms	Best enhancers identified for neuron type from Janelia GAL4 library	Best enhancers identified for neuron type from Vienna tiles (VT) GAL4 library	FlyCircuit.tw - Single cell examples for neuron type	Clusters corresponding to optic glomerulus or tract associated with a VPN			
					C (Janelia FlyLight dataset)	C' (Vienna Tiles dataset)	C'' (Janelia FlyLight dataset, 2nd run)	C''' (Vienna Tiles dataset, 2nd run)
LC04	Col A (Mu et al., 2012; Strausfeld & Okamura, 2007; Strausfeld and Hausen, 1977)	GMR26G09, GMR47H03	VT042758, VT046005	Cha-F-000138, Cha-F-200257, Gad1-F-300256	C33, C21, C15, C25	C'26, C'39	C''02, C''17	C'''24
LC06	S4 (Fischbach and Lyly-Hünerberg, 1983)	GMR41C07, GMR22A07	VT006549, VT009855	Cha-F-000039, Gad1-F-400244, Gad1-F-200326	C57	C'27	C''48	C'''42
LC09	S4 (Fischbach and Lyly-Hünerberg, 1983)	GMR71C02, GMR14A11	VT014209, VT005102, VT027704	Cha-F-000028, Gad1-F-700145, Gad1-F-200274	C32, C14	C'59, C'60	C''52, C''56, C''35	C'''02
LC10	S3 (Fischbach and Lyly-Hünerberg, 1983)	GMR22D06, GMR35D04	VT021760, VT043920	Gad1-F-100080, Cha-F-300390, fru-F-800100	C22, C09, C19	C'32, C'55, C'48, C'29	C''03, C''54, C''49, C''06	C'''33, C'''34, C'''50
LC11	L1CN (Mu et al., 2012)	GMR23D02, GMR87B04, GMR51F09, GMR22H02	VT004968, VT008647, VT004967	Cha-F-000153, Cha-F-200132, Gad1-F-300060	C07, C45	C'18	C''32. C''30	C'''16
LC12		GMR59B10, GMR35D04, GMR19G01	VT062247, VT040919	Cha-F-000124, Cha-F-000015, VGlut-F-000056, VGlut-F-400347	C26, C05	C'06	C''45	C'''39, C'''13
LC13		GMR50C10, GMR14A11	VT057283, VT025771	Cha-F-000255, Cha-F-100003, Gad1-F-100040	C46	C'51	C''26, C''01	C'''58
LC14	DC neurons (Hassan et al., 2000)	GMR21H10, GMR12F01, GMR58H11	VT037804	Cha-F-400228, Cha-F-400231, Gad1-F-300016	x	C'03	C''34	C'''08
LC15		GMR42H06, GMR24A02	VT014207, VT047878, VT012320	Cha-F-000361, Cha-F-100351	C28	C'44	C''33, C''21	C'''41, C'''40
LC16		GMR32D04, GMR25G03	VT061079, VT025771	Gad1-F-100202, Cha-F-000316, fru-F-000032, VGlut-F-000603	C37, C03	C'40, C'27	C''47	C'''49
LC17		GMR21B04, GMR65C12	VT034259, VT033301	Cha-F-100017, Cha-F-000004, Gad1-F-000025	C23, C26, C01	C'35, C'38, C'58	C''08, C''45	C'''38, C'''29, C'''35, C'''11, C'''39, C'''60, C'''12
LC18		GMR92B11	VT008183	5-HT1B-F-500016, Cha-F-000333, fru-F-200061, Gad1-F-300054	C29, C02	C'01	C''07, C''53	C'''37, C'''44
LC20		GMR17A04, GMR71G09	VT025718	VGlut-F-200564, VGlut-F-700163, Gad1-F-200101	C43	x	C''10	x
LC21		GMR85F11, GMR25A07	VT014960	Gad1-F-400102, Cha-F-300208	C40, C28, C07	C'18	C''30, C''40	C'''40, C'''16
LC22/LPLC4		GMR24A05	VT058688	LC22: Gad1-F-900022, Cha-F-600134, VGlut-F-500700<br>LPLC4: Gad1-F-200058, Cha-F-200302, Cha-F-200028	C16	C'42, C'19	C''57	C'''14
LC24		GMR20G09	VT038216	Cha-F-000283, Cha-F-200073, Cha-F-400116	C37	C'40	C''47	C'''10
LPLC1	LPL2CN (Mu et al., 2012)	GMR36B06, GMR12G03	VT007767	Cha-F-200219, Cha-F-300035, Gad1-F-400140	C18, C44, C25	C'07	C''25	C'''30
LPLC2		GMR75G12, GMR12E04	VT007194, VT049479	Gad1-F-000300, Cha-F-100287, Cha-F-300111	C44	C'21	C''25	C'''06, C'''30
LPLC3		GMR9C11, GMR49A05	VT044492, VT062624	Cha-F-100027, Cha-F-300004, Gad1-F-200099, fru-F-500009	C35, C55, C20, C30	C'46, C'05, C'09	C''59, C''13, C''19	C'''28, C'''14
LPC1		GMR37G12, GMR77A06, GMR81A05, GMR20A09 (subset)	VT046005	VGlut-F-700361, Cha-F-000272, fru-F-000101	C04, C30, C20	C'05	C''12, C''59, C''19	C'''46
MC61	LC10c (Otsuna & Ito, 2006)	GMR53B08	VT002072, VT021203	Gad1-F-400023, Cha-F-300285, Cha-F-200026,	C56	C'34, C'10	C''49	C'''17
MC62		GMR78G04, GMR85C01	VT062624	none identified	C48	C'56	C''05	x
MC63	VPN-MB1? (Vogt et al., 2016)!	GMR72C11	VT022290, VT008183, VT017001	Cha-F-200103	C42, C48	C'25, C'56	C''04, C''11, C''05	C'''55
Lat		GMR16G04, GMR13E10, GMR85G07, GMR39F04	VT045604, VT014963, VT033613	TH-F-200107, Trh-F-100019, TH-F-100004, Cha-F-300333	C50, C42	C'30, C'52, C'56, C'57	C''04	C'''55

Table S1. (data related to Figure 4): Table with VPN, Clusters, Driver lines, Flycircuit IDs.

Note: MC63 may be synonymous with VPN-MB1 [S3], which was published while this study was under review.

Supplemental Experimental Procedures

Thresholding, Dice similarity, k-Medoids, and Hierarchical Agglomeration

GAL4 expression patterns were transformed into a binary representation in two steps. First, the image is thresholded and second, morphological opening (dilation of the erosion by a 3x3x3 structuring kernel) is applied to reduce clutter. The threshold was chosen so that the resulting mask yielded 1% stained voxels. This simple heuristic was more reliable for the datasets tested compared to other standard automatic thresholding methods.

From the binarized images, the set of expressing lines was assembled for each voxel. Similarity between voxels based on the respective expression set from voxel A and the set from voxel B is computed using Dice's coefficient as $s = \frac{2|A \cap B|}{|A| + |B|}$ where ∩ denotes intersection and |*x*| denotes the number of elements in set *x*. To decrease the effects of registration error and image acquisition noise and to increase the speed of subsequent processing steps, we binned the original image voxel data into larger voxels, using a 3x3x3 nearest-neighbor downsampling. Analysis was performed on specific brain regions (e.g. antennal lobe or oVLNP) defined by a 3D brain atlas of neuropils (included in the supplemental data). Voxels in the bounding cube but not in the defined neuropil were excluded. The k-medoids algorithm [S1] was run in Julia 0.4.0 using JuliaStats Clustering 0.5.0 (see Supplementary file 1). The *k*-medoids was performed on Dice dissimilarity (1-*s*). To agglomerate the medoids, we used the fastcluster package [S2] with Python 2.7.10 using average linkage with metric distance $\sqrt{1-s}$ between medoids.

Initial clustering was performed on a distance matrix found as follows. For each voxel within the analyzed brain region (e.g. antennal lobe or lateral protocerebrum), we calculated the set of driver lines for which GFP expression was higher than a threshold. We used the Dice coefficient (a measure of overlap, see above) to quantify expression similarity between each possible pair of *n* voxels. This *n* x *n* distance matrix was used to group voxels into clusters of similar expression using *k*-medoids clustering, a standard clustering technique (Figure 1A, see Experimental Procedures for details). Clustering with other standard algorithms such as mini-batch *k*-means gave qualitatively similar results, and we focus here on *k*-medoids only for convenience. As typical for clustering algorithms, one parameter controls the number of clusters, and in our case we chose several different values for *k* and evaluated results for different choices and in each of the two independent datasets. Every voxel in the analysis is assigned to exactly one cluster. Neither manual inspection nor calculation of a metric designed to measure clustering repeatability, adjusted Rand index (Figure S1A), showed an obvious optimal value for *k*. Therefore, we chose a value of *k* equal 60 as a number which appeared to provide sufficiently many

clusters to capture important structures at a small scale without producing an overwhelming number. The result of the initial clustering algorithm is the assignment of each voxel in the input brain region to one of *k* clusters. The second major step, hierarchical clustering, took the cluster centers from the first step and agglomerated these 'singletons' into 2*k*-1 clusters.

Evaluating repeatability of clustering

As discussed above, automatic calculation of a measure of repeatability (adjusted Rand index, Figure S1A) found no obvious optimum value of *k* used in the initial clustering step. Therefore, we sought to gain a more biologically meaningful sense of consistency across multiple runs of the algorithm for *k=60* by comparing visually the results of manual and automatic segmentations. We did this for the oVLNP with each of four different clustering runs, two from each dataset (Figure S3). The results show that, despite different random number initialization seeds, most optic glomeruli have a strong correspondence with a singleton cluster across repeated runs of the algorithm within and across the two datasets (Vienna Tiles and Janelia FlyLight). This indicates substantial biologically meaningful repeatability within and between datasets at the first clustering step, which agglomeration then structures hierarchically.

Supplemental References

S1. Kaufman, L., and Rousseeuw, P. J. (1987). Clustering by Means of Medoids. In *Statistical Data Analysis Based on the L1 Norm and Related Methods.*

S2. fastcluster: Fast Hierarchical, Agglomerative Clustering Routines for R and Python *Journal of Statistical Software* https://www.jstatsoft.org/article/view/v053i09.

S3. Vogt, K., Aso, Y., Hige, T., Knapek, S., Ichinose, T., Friedrich, A. B., Turner, G. C., Rubin, G. M., and Tanimoto, H. (2016). Direct neural pathways convey distinct visual information to Drosophila mushroom bodies. *eLife 5*, e14009.
